# Fibin regulates cardiomyocyte hypertrophy and causes protein-aggregate-associated cardiomyopathy *in vivo*

**DOI:** 10.3389/fmolb.2023.1169658

**Published:** 2023-06-05

**Authors:** Matthias Petersen, Nesrin Schmiedel, Franziska Dierck, Susanne Hille, Anca Remes, Frauke Senger, Inga Schmidt, Renate Lüllmann-Rauch, Oliver J. Müller, Derk Frank, Ashraf Y. Rangrez, Norbert Frey, Christian Kuhn

**Affiliations:** ^1^ Department of Internal Medicine III, University Medical Center of Schleswig-Holstein, Campus Kiel, Kiel, Germany; ^2^ DZHK (German Centre for Cardiovascular Research), Partner Site Hamburg/Kiel/Lübeck, Kiel, Germany; ^3^ Department of Anatomy, Christian-Albrechts-University, Kiel, Germany; ^4^ Department of Cardiology, Angiology and Pneumology, Heidelberg University Hospital, Heidelberg, Germany; ^5^ DZHK (German Centre for Cardiovascular Research), Partner Site Heidelberg/Mannheim, Heidelberg, Germany

**Keywords:** fibin, cardiac z-disc, cardiac hypertrophy, SRF signalling, NFAT signalling, protein aggregate associated cardiomyopathy

## Abstract

Despite the identification of numerous molecular pathways modulating cardiac hypertrophy its pathogenesis is not completely understood. In this study we define an unexpected role for Fibin (“fin bud initiation factor homolog”) in cardiomyocyte hypertrophy. Via gene expression profiling in hypertrophic murine hearts after transverse aortic constriction we found a significant induction of Fibin. Moreover, Fibin was upregulated in another mouse model of cardiac hypertrophy (calcineurin-transgenics) as well as in patients with dilated cardiomyopathy. Immunoflourescence microscopy revealed subcellular localization of Fibin at the sarcomeric z-disc. Overexpression of Fibin in neonatal rat ventricular cardiomyocytes revealed a strong anti-hypertrophic effect through inhibiting both, NFAT- and SRF-dependent signalling. In contrast, transgenic mice with cardiac-restricted overexpression of Fibin developed dilated cardiomyopathy, accompanied by induction of hypertrophy-associated genes. Moreover, Fibin overexpression accelerated the progression to heart failure in the presence of prohypertrophic stimuli such as pressure overload and calcineurin overexpression. Histological and ultrastructural analyses surprisingly showed large protein aggregates containing Fibin. On the molecular level, aggregate formation was accompanied by an induction of the unfolded protein response subsequent UPR-mediated apoptosis and autophagy. Taken together, we identified Fibin as a novel potent negative regulator of cardiomyocyte hypertrophy *in vitro*. Yet, heart-specific Fibin overexpression *in vivo* causes development of a protein-aggregate-associated cardiomyopathy. Because of close similarities to myofibrillar myopathies, Fibin represents a candidate gene for cardiomyopathy and Fibin transgenic mice may provide additional mechanistic insight into aggregate formation in these diseases.

## 1 Introduction

Cardiovascular diseases like hypertension, ischaemic or valvular heart disease or hypertrophic cardiomyopathy are associated with cardiac hypertrophy. The initially compensatory hypertrophic response may turn maladaptive over the time resulting in an increased risk for heart failure, arrhythmias and sudden cardiac death (Levy et al., 1990). Consequently, cardiac hypertrophy has raised attention as a potential therapeutic target in cardiovascular disease. In the past decades, numerous signalling pathways have been identified that mediate cardiac hypertrophy. Especially the cardiac z-disc has been defined as a nodal point in hypertrophic signalling (Frank et al., 2006).

Located at the lateral boundaries of the sarcomere, the cardiac z-disc serves as an anchoring site for the thin filaments of adjacent sarcomeres. Via costameres, an assembly of peripheral z-disc proteins and subsarcolemmal proteins, the cardiac z-disc is linked to the sarcolemma. This unique localization of the cardiac z-disc is responsible for its important role in force transmission in cardiac and skeletal muscle. However, the z-disc does not play a mere mechanical role. Many proteins located to the z-disc are members of various signalling cascades.

One central player in hypertrophic signalling at the cardiac z-disc is the serine-threonin phosphatase calcineurin (Parra and Rothermel, 2017). Calcineurin is a heterodimer consisting of a catalytic subunit CnA with the isoforms CnAα, CnAβ, and CnAγ as well as a regulatory subunit CnB with the isoforms CnB1 and CnB2 (Wilkins and Molkentin, 2004). Once activated by calcium ions and a complex of calcium and calmodulin, calcineurin dephosphorylates transcription factors of the nuclear factor of activated T-cell (NFAT) family and promotes their translocation into the nucleus. This in turn activates the expression of hypertrophy-associated genes like *Nppa*, *Nppb* and *Myh7*. While cardiac-specific overexpression of constitutively active calcineurin in mice leads to severe cardiac hypertrophy and heart failure (Molkentin et al., 1998), a homozygous knockout of the catalytic subunit CnAβ in mice diminishes the hypertrophic response to pressure overload or agonists (Bueno et al., 2002). Mice with a total loss of calcineurin activity through lack of the regulatory subunit CnB1 are not viable (Graef et al., 2001). Recently, numerous proteins have been identified to modulate cardiac hypertrophy by interaction with calcineurin at the cardiac z-disc (Heineke and Ritter, 2012).

Besides NFAT, the serum response factor (SRF) is another transcription factor with implications in hypertrophic signalling. Together with several cofactors, SRF controls the expression of many mitogen-responsive and muscle-specific genes (Posern and Treisman, 2006). Basically, SRF-dependent gene expression is activated by two different signalling pathways. On the one hand, mitogen activated protein (MAP) kinases phosphorylate members of the ternary complex factor (TCF) family of Ets domain transcription factors which promotes the formation of a ternary complex with SRF and DNA (Treisman, 1994). On the other hand, SRF-dependent gene expression is activated through binding to members of another family of transcriptional cofactors, the myocardin-related transcription factors (MRTFs). The activity of MRTFs is regulated by a signalling pathway involving Rho-family GTPases and monomeric actin (Posern and Treisman, 2006). Stimulation of Rho-GTPases leads to polymerization of G-actin to F-actin. This results in a dissociation of MRTFs from G-actin and their translocation to the nucleus. Free MRTFs in the nucleus can associate with SRF and bind to CArG boxes to promote target gene expression (Posern and Treisman, 2006).

We aimed to identify novel genes involved in cardiac hypertrophy by analysing the gene expression profile of hearts from mice that had undergone transverse aortic constriction. Microarray expression profiling identified a set of differentially regulated genes. Besides many established genes including *Myh7*, *Nppa* and *Nppb*, we could show a 4.5-fold (*p* < 0.001) upregulation of *Fibin* (fin bud initiation factor homolog).

Fibin has first been described as a novel protein with a role in the embryogenesis of zebrafish, as knockdown of Fibin in zebrafish using morpholino antisense oligonucleotides prevented the initiation of the pectoral fin bud (Wakahara et al., 2007). Fibin is an evolutionarily conserved protein with no structural homology to any known protein. Besides its expression in several embryonic stages in zebrafish, Fibin is also expressed in several adult mouse tissues including skeletal muscle and heart indicating a role of Fibin not only in embryonic stages but also in adult tissue (Lakner et al., 2011). Interestingly, Fibin is upregulated in the right ventricles of rats during chronic pulmonary embolism (Zagorski et al., 2009). However, so far very little is known about its function in mammalian cells.

Here we report for the first time a distinct function of Fibin in the context of cardiomyocyte hypertrophy and cardiomyopathy.

## 2 Materials and methods

### 2.1 Microarray

Adult female C57BL/6N mice underwent transverse aortic constriction (TAC, *n* = 7) for a period of 2 weeks. Sham operated mice served as a control group (*n* = 8). After 2 weeks banded animals had developed significant hypertrophy (Heart weight vs body weight: sham 5.3 ± 0.4 mg/g, TAC 8.2 ± 1.3 mg/g, *p* < 0.001) which was accompanied by impaired left ventricular function (fractional shortening: sham 34.8% ± 9.6%, TAC 13.8% ± 7.4%, *p* < 0.001). Microarray expression profiling (Agilent SurePrint G3 Mouse GE 8 × 60K) was performed with RNA derived from left ventricles of banded and sham operated mice to identify a set of differentially regulated genes.

### 2.2 Cloning of Fibin overexpression constructs

Full-length Fibin was cloned using mouse heart cDNA as a template and the following primers: mFibin_attB1: 5′-GCT​GGC​ACC​ATG​GTG​TTC​CCG​AAG-3´; 5′-GCT​GGG​TCG​CCT​TAG​CCT​GTC​TTC​TG-3´. The PCR product was recombined into a pDONR221 Gateway vector using the Gateway cloning technology (Thermo Fisher Scientific Inc.). Adenoviruses coding for overexpression constructs were generated using the ViraPower Adenoviral Expression System (Thermo Fisher Scientific Inc.) according to the manufacturer’s protocol. A β-galactosidase V5-encoding adenovirus (AdlacZ, Thermo Fisher Scientific Inc.) served as control virus.

### 2.3 Cloning of synthetic miRNA against Fibin

Synthetic miRNAs against Fibin for knockdown experiments in NRVCMs were designed using Invitrogen’s BLOCK-iT RNAi Designer and cloned into a pcDNA 6.2-GWmiR vector according to the manufacturer’s protocol (Thermo Fisher Scientific Inc.). A negative control plasmid, which is predicted not to target any known mammalian gene, served as a control. The constructs were recombined into the pDonR221 plasmid via Gateway cloning technology (Thermo Fisher Scientific Inc.). Adenoviruses coding for synthetic microRNAs were generated using the ViraPower Adenoviral Expression System (Thermo Fisher Scientific Inc.) following the manufacturer’s instructions.

Template sequence for cloning of synthetic miRNA against Fibin:

5′-TGC​TGA​AGA​GTG​AGG​CTC​AGC​AAG​CTG​TTT​TGG​CCA​CTG​ACT​GAC​AGC​TTG​CTG​CCT​CAC​TCT​T -3′

5′- CCT​GAA​GAG​TGA​GGC​AGC​AAG​CTG​TCA​GTC​AGT​GGC​CAA​AAC​AGC​TTG​CTG​AGC​CTC​ACT​CTT​C -3′

### 2.4 Isolation and culture of neonatal rat ventricular cardiomyocytes

Hearts from 1- to 2-day-old Wistar rats (Charles River Laboratories) were excised and minced in ADS buffer (120 mmol/L NaCl, 20 mmol/L HEPES, 8 mmol/L NaH2PO4, 6 mmol/L glucose, 5 mmol/L KCl, 0.8 mmol/L MgSO4, pH 7.4). After a series of digestion steps by using an enzymatic solution containing collagenase type II (0.5 mg/mL, Worthington) and pancreatin (0.6 mg/mL, Sigma Aldrich) in sterile ADS buffer, a Percoll (GE Healthcare) gradient centrifugation step was carried out to separate fibroblasts from cardiomyocytes. Neonatal rat ventricular cardiomyocytes (NRVCMs) were cultured in Dulbecco’s modified Eagle’s medium (DMEM, Gibco) containing 10% FCS (Capricorn Scientific), 100 U/L penicillin, 100 μg/mL streptomycin (Gibco) and 2 mM L-glutamine (Gibco). Cells were infected in serum-free medium after 24 h. 24 h after infection medium was replaced and cells were stimulated with 100 μM phenylephrine (Sigma-Aldrich) for 48 h.

### 2.5 Immunoblotting

NRVCMs were harvested in lysis buffer and lysed by three freeze and thaw cycles. For protein isolation from murine hearts, tissue was shredded in lysis buffer. To remove cell debris a centrifugation step (12,000 g, 20 min, 4°C) was carried out. Protein concentration was determined photometrically on an Infinite m200 PRO system (Tecan) by using the Bradford protein assay kit according to the manufacturer’s manual (Bio-Rad). Protein samples were resolved by 10% SDS-PAGE and transferred onto a polyvinylidenefluoride (PVDF) membrane. After blocking in 5% dry-milk in Tris-buffered saline with Tween20 (TBS-T), the incubation with the target-specific primary antibody was carried out overnight at 4°C, followed by application of a suitable horseradish peroxidase-coupled secondary antibody (Supplementary Table S1). For visualization of protein bands, an ECL Select chemiluminescence kit (GE Healthcare) and the Flourchem Q imaging system (Biozym) was used. Quantitative densitometric analysis was carried out with ImageJ/Fiji version 1.46. Tubulin, GAPDH or whole protein content stained by Ponceau was used for normalization.

### 2.6 RNA isolation and quantitative real-time PCR

Using QIAzol lysis reagent (Qiagen), total RNA was isolated from NRVCMs according to the manufacturer’s instructions. After DNase I-digestion and purification, 1 μg of total RNA was transcribed into cDNA using the Superscript III first strand cDNA synthesis kit (Thermo Fisher Scientific Inc.). Real Time PCR measurements were carried out in CFX96 real-time cycler (Biorad) with the EXPRESS SYBR GreenER Reagent (Thermo Fisher Scientific Inc.) or iQ Multiplex Powermix (Bio-Rad).

### 2.7 Reporter gene assays

NRVCMs were infected with a combination of viruses coding for Fibin (25 moi, 50 moi or 100 moi), RhoA (10 moi) or a constitutively active variant of calcineurin (50 moi) along with SRF-RE-luc (10 moi) or NFAT-RE-luc (10 moi), carrying a firefly luciferase. AdlacZ served as a control virus and to maintain equal virus loads. 36 h after infection cells were harvested in passive lysis buffer. A dual luciferase reporter assay (Promega) was performed following the manufacturer’s instructions. Measurements were normalized to total protein concentration. Protein concentration was determined photometrically on an Infinite m200 PRO system (Tecan) by using the Bradford protein assay kit according to the manufacturer’s manual (Bio-Rad).

### 2.8 Immunoflourescence microscopy

NRVCMs and mouse tissue cryosections were fixed with 4% paraformaldehyde in PBS for 10 min at room temperature. After permeabilization and blocking with 2.5% BSA and 0.1% Triton X-100 in PBS for 1 h at room temperature, the primary antibody was applied and the slides were incubated at 4°C overnight. Flourescent-dye conjugated secondary antibodies were used at a dilution of 1:500 for 1 h at room temperature (Supplementary Table S1). Nuclei staining was performed simultaneously with DAPI (4′,6′-diamidino-2-phenylindole, Vector Laboratories). Finally, slides were mounted with FluorPreserve reagent (Merck). Images were captured on a BZ-9000-E HS all-in-one fluorescence microscope (Keyence) or a Zeiss LSM-800 confocal microscope (Axio Observer. Z1/7 microscope).

### 2.9 Cell surface area measurements in NRVCMs

5 × 5 × 5 (x y z) pictures were taken on a BZ-9000-E all-in-one fluorescence microscope (Keyence) at × 20 magnification and merged. The cell size was measured using Keyence’s HybridCellCount software module in fluorescence intensity single-extraction mode as described earlier (Rangrez et al., 2013).

### 2.10 Cell surface area measurements in heart tissue sections

After overnight fixation in 2% paraformaldehyde, murine hearts were cut on level of papillary muscle and embedded in optimal cutting temperature (OCT) medium (Tissue-Tek, Sakura). Heart sections were stained with FITC-conjugated lectin from Triticum vulgaris (Sigma). Images were captured on a BZ-9000-E HS all-in-one fluorescence microscope (Keyence) at × 4 magnification (CFI Plan Apochromatic λ × 4 objective, NA 0.20; Nikon). The cell size was measured using Keyence’s HybridCellCount software in non-fluorescence intensity single extraction mode.

### 2.11 Quantification of myocardial fibrosis

Heart sections were subjected to Masson trichrome staining and images were taken on BZ-9000-E HS all-in-one fluorescence microscope (Keyence) at × 4 magnification (CFI Plan Apochromatic λ × 4 objective, NA 0.20; Nikon). Quantification of fibrosis was performed by a novel machine learning-based tool (FibroSoft) as described elsewhere (Remes et al., 2023).

### 2.12 Electron microscopy

Electron microscopy was carried out as described previously (Hille et al., 2016). The heart was perfused with 1% procaine in 0.1 M PBS and fixed with 6% glutaraldehyde in 0.1 M PBS. Tissue blocks were post-fixed with 2% osmium tetroxide and embedded in araldite. Ultrathin sections were processed with uranyl acetate and lead citrate and viewed with Zeiss EM 900 microscope.

### 2.13 Generation of Fibin transgenic mice

Murine Fibin-cDNA was cloned into an α-MHC-promoter bearing plasmid that carries a c-terminal flag-tag and a human growth hormone (hGH) poly (A) + signal (Gulick et al., 1991a). In cooperation with the Max Planck Institute of Molecular Cell Biology and Genetics Dresden, the plasmid construct was microinjected into blastocyst pronuclei with C57BL6/N background which were transferred in Blb/c pseudopregnant females mice. Several transgenic founders were obtained and three different mouse lines were established based on the protein level of Fibin. Genotyping was carried out by PCR using primers for Fibin 5′-GGAGACACTGGACATCTCTG-3′and the hGH poly (A) + signal 5′-CCT​CTC​CTG​GCC​CTG​GAA​GTT​G-3’.

### 2.14 Animal experiments

Animal handling was performed according to the guidelines from Directive 2010/63/EU of the European Parliament on the protection of animals used for scientific purposes and according to the institutional guidelines of the University Medical Center Schleswig-Holstein as well as of the state of Schleswig-Holstein, Germany. All animal experiments were approved by the state of Schleswig-Holstein, Germany [References: 242-53853/2016 (104-8/16)].

To assess cardiac function, mice were anaesthetized by inhalation of isoflurane (1.5% v/v) and echocardiography was performed. After echocardiography mice were killed by cervical dislocation and hearts were analysed by various histological and molecular biological techniques.

### 2.15 Echocardiography

Echocardiography was carried out using the Vevo 1100 imaging system with a MS400 cardiovascular probe (18–38 MHz, VisualSonics). After anaesthesia with isoflurane (1.5% v/v), mice were placed onto a heating pad and an ECG was connected. During the procedure mouse body temperature was maintained at 37°C and the heart rate was controlled continuously. Parameters were measured by M-Mode in a short axis view at the level of the papillary muscles.

### 2.16 Transverse aortic constriction

Transverse aortic constriction was performed in transgenic mice and their wildtype littermates at the age of 8 weeks. Before surgery the animals received analgesia (buprenorphine 0.1 mg/kg body weight) and were anesthetized with isoflurane (1.5% v/v). During the procedure mice were orally intubated and ventilated at 120 breaths per minute with 0.2 mL tidal volume. After lateral thoracotomy through the second intercostal space, the transverse aorta was ligated between the brachiocephalic and left carotid artery against a 26 G needle. After removing the needle the chest was closed. Sham operated mice underwent the same operation except ligation of the aortic arch and served as a control. Mice were examined and killed 2 weeks after surgery.

### 2.17 Human tissue processing

Left ventricular myocardial tissue was taken from explanted hearts with end-stage heart failure. The explanted hearts were directly acquired in the operating room during surgical procedure and immediately put in pre-cooled cardioprotective solution (Custodiol, Dr. Franz Köhler Chemie). Myocardial samples for Western blot analysis were snap frozen (−80°C) immediately after excision. Healthy donor hearts that could not be transplanted for technical reasons were used as controls. All procedures were conducted following the Declaration of Helsinki and in compliance with the local ethics committee.

### 2.18 Statistical analysis

Results are presented as means ± standard error of the mean (S.E.) unless stated otherwise. Statistical analyses were performed using two-tailed Student’s *t* test or two-way ANOVA followed by Student-Newman-Keuls *post hoc* tests. Normality and equal variance testing was performed before ANOVA. Statistical analysis of the gene reporter assays were carried out by using one-way ANOVA on ranks (Kruskal-Wallis test) followed by Dunn’s *post hoc* tests. *p* values < 0.05 were considered statistically significant.

## 3 Results

### 3.1 Fibin is differentially regulated in murine models of cardiac hypertrophy and in patients with dilated cardiomyopathy

**FIGURE 1:**
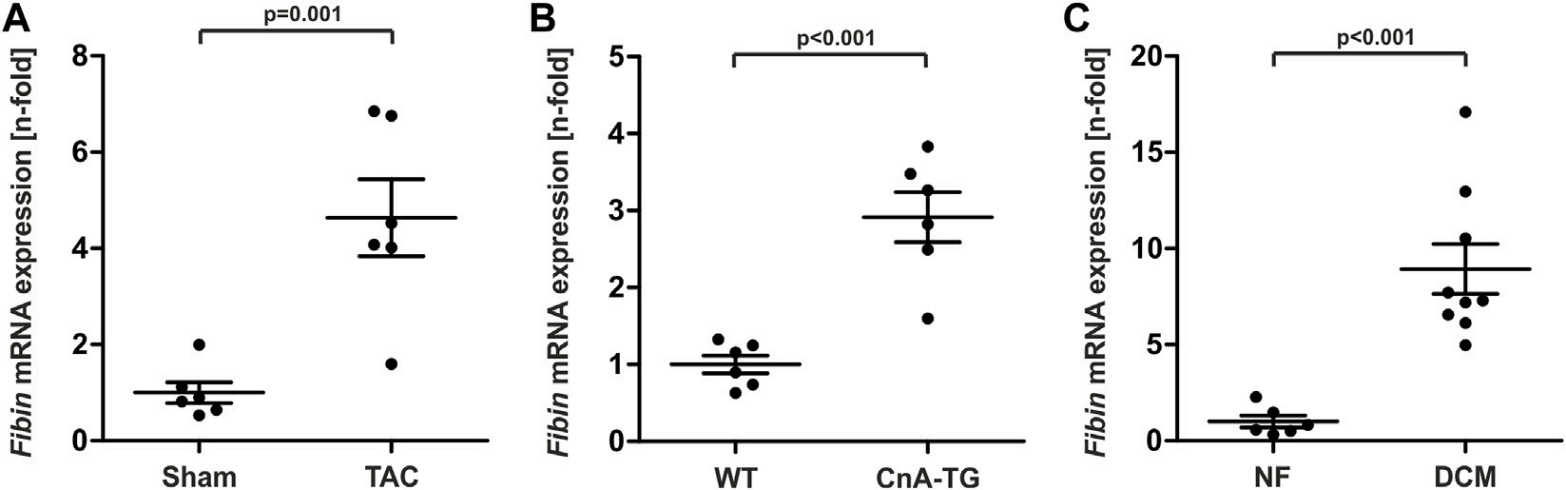
Fibin is upregulated in murine models of cardiac hypertrophy and in patients with dilated cardiomyopathy (DCM). *Fibin* mRNA expression in **(A)** mice after transverse aortic constriction (TAC), **(B)** in calcineurin-transgenic mice (CnA-TG) (*n* = 6 per group), and **(C)** in patients with DCM (*n* = 9, vs human non failing hearts NF, *n* = 6) detected by qPCR. The expression of Fibin was measured 2 weeks after TAC. The CnA-TG and their littermates were killed at the age of 2 months. All values are expressed as mean ± SEM. Significance was assessed by two-tailed Student´s *t*-test.

Using quantitative real time PCR (qPCR) we detected induction of *Fibin* in hypertrophic hearts from mice after transverse aortic constriction (TAC) and from transgenic mice that overexpresses constitutively active calcineurin (Figures 1A,B). *Fibin* is also upregulated in patients with dilated cardiomyopathy (DCM) (Figure 1C).

Because of this robust induction we wondered whether Fibin might play a role in cardiac hypertrophy. To elucidate the effect of Fibin on cardiomyocyte hypertrophy we investigated the influence of altered Fibin expression on isolated neonatal rat ventricular cardiomyocytes (NRVCMs) in the absence or presence of the pro-hypertrophic agent phenylephrine (PE).

### 3.2 Overexpression of Fibin effectively inhibits cardiomyocyte hypertrophy induced by phenylephrine

**FIGURE 2:**
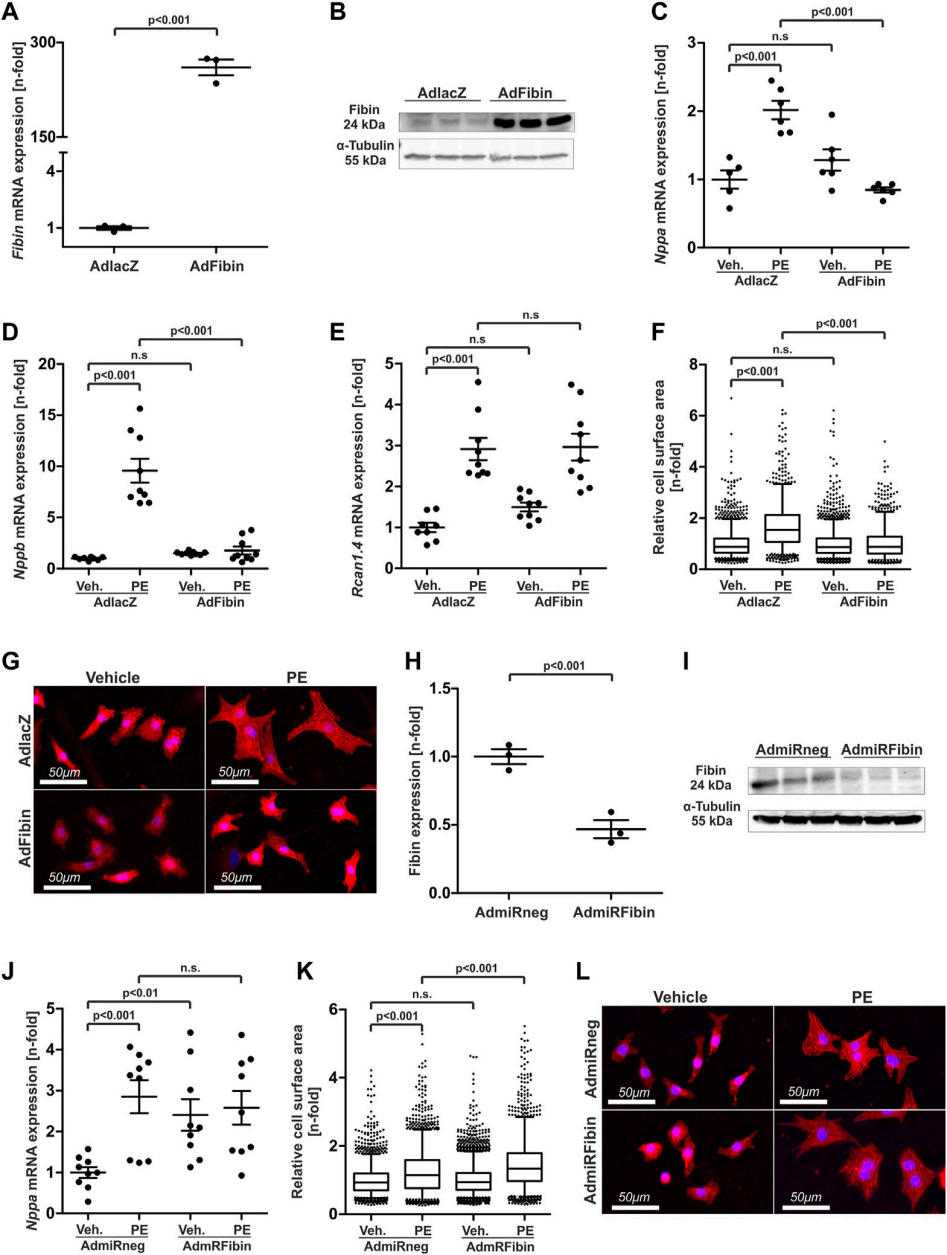
Adenoviral overexpression of Fibin in NRVCMs inhibits cardiomyocyte hypertrophy induced by phenylephrine while knockdown of Fibin induces hypertrophic signalling. **(A)** Overexpression of Fibin in neonatal rat ventricular cardiomyocytes (NRVCMs) at the mRNA- (*n* = 3 per group, one experiment, two-tailed Student´s t-test) and **(B)** protein level. **(C)** *Nppa* expression in NRVCMs after overexpression of Fibin and/or stimulation with phenylephrine (PE) (*n* = 6 per condition, two independent experiments, two-way ANOVA followed by Student-Newman-Keuls *post hoc* test). **(C)** *Nppa* mRNA expression (*n* = 6 per condition, two independent experiments, two-way ANOVA followed by Student-Newman-Keuls *post hoc* test), **(D)** *Nppb* mRNA expression (*n* = 8–9 per condition, three independent experiments, two-way ANOVA followed by Student-Newman-Keuls *post hoc* test) and **(E)** *Rcan1.4* mRNA expression (*n* = 8–9 per condition, three independent experiments, two-way ANOVA followed by Student-Newman-Keuls *post hoc* test) in NRVCMs after overexpression of Fibin and/or stimulation with phenylephrine (PE), detected by qPCR. **(F)** Cell surface area (CSA) of NRVCMs under Fibin overexpression and/or PE stimulation (*n* > 1000 per condition, two independent experiments, two-way ANOVA followed by Student-Newman-Keuls *post hoc* test). **(G)** Representative images of β-galactosidase (50 moi) and Fibin (50 moi)-overexpressing cardiomyocytes stained with an α-actinin antibody. Red: α-actinin, blue: DAPI. **(H)** Knockdown of Fibin in NRVCMs on mRNA- (*n* = 3, one experiment, two-tailed Student´s *t*-test) and **(I)** on protein level. **(J)** *Nppa* mRNA expression in NRVCMs after knockdown of Fibin and/or PE stimulation (*n* = 9 per condition, three independent experiments, two-way ANOVA followed by Student-Newman-Keuls *post hoc* test). **(K)** CSA of NRVCMs after knockdown of Fibin and/or PE stimulation (*n* > 1800 per condition, three independent experiments, two-way ANOVA followed by Student-Newman-Keuls *post hoc* test). **(L)** Representative images of miRneg (200 moi) and miRFibin (200 moi) overexpressing cardiomyocytes stained with an α-actinin antibody. Red: α-Actinin, blue: DAPI. Boxplots with mean and interquartile range, whiskers show 5^th^ and 95^th^ percentiles, dots represent outliers from this range.

Infection of NRVCMs with an adenovirus coding for Fibin led to a significant overexpression at the mRNA and protein level (Figures 2A,B). NRVCMs infected with an adenovirus coding for β–galactosidase (AdlacZ) served as a control. Fibin overexpression dramatically diminished the PE-mediated induction of members of the hypertrophic gene program like *Nppa* (Figure 2C) and *Nppb* (Figure 2D). The expression of *Rcan1.4*, another marker of hypertrophy was not affected by Fibin overexpression (Figure 2E). This effect was also detectable on cellular level. While treatment with PE (100 μmol/L) led to a significant increase of cell surface area (CSA) of control cardiomyocytes, adenoviral overexpression of Fibin effectively inhibited this cellular hypertrophy induced by PE (Figures 2F,G).

Given this strong anti-hypertrophic effect of Fibin, it was our aim to examine, if a loss of function of Fibin is sufficient to induce cardiomyocyte hypertrophy. Knockdown experiments were carried out by adenoviral gene transfer of a synthetic miRNA against Fibin (AdmiRFibin) into NRVCMs. An adenovirus coding for a miRNA without any known target served as a control (AdmiRneg). The expression of miRFibin in NRVCMs led to relevant downregulation of Fibin on mRNA and protein level (Figures 2H,I). While the knockdown of Fibin induced the expression of members of the hypertrophic gene program like *Nppa* (Figure 2J), it was not sufficient to induce cellular hypertrophy. However, knockdown of Fibin caused an exaggerated hypertrophic response to PE-stimulation, displayed by an increase in CSA compared to stimulated control cells (+17.1%, Figure 2K–L). In contrast, Fibin knockdown and PE-stimulation showed no additive effect on the expression of *Nppa* (Figure 2J).

### 3.3 Fibin co-localizes with α-actinin at the sarcomeric z-disc and inhibits SRF- and NFAT-dependent gene expression

**FIGURE 3:**
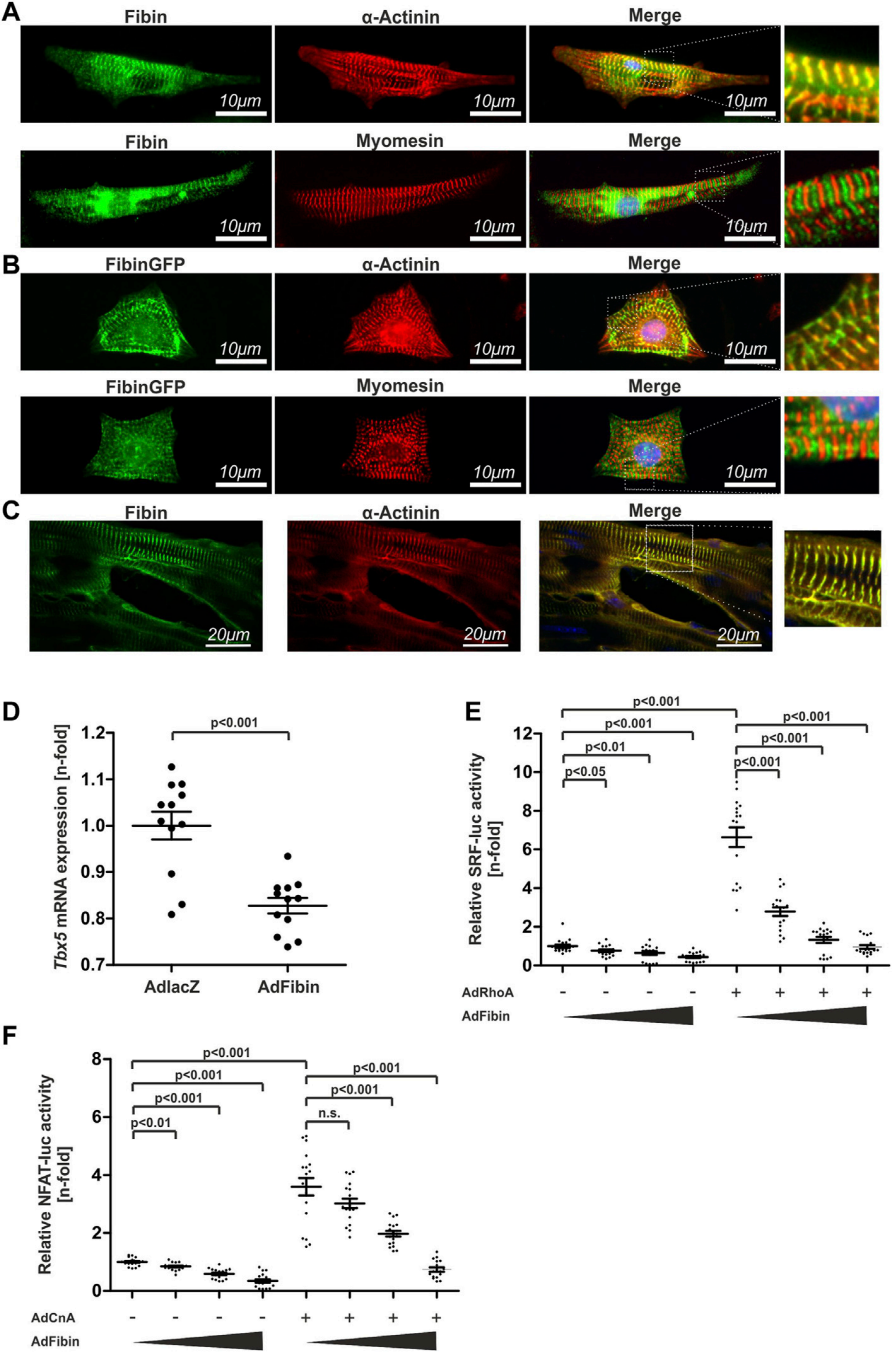
Fibin co-localizes with α-actinin at the sarcomeric z-disc and effectively inhibits NFAT- and SRF-dependent gene expression. **(A)** Co-staining of Fibin and α-actinin or Myomesin in neonatal rat ventricular cardiomyocytes (NRVCMs) using specific antibodies. **(B)** Overexpression of a Fibin-GFP fusion protein in NRVCMs and co-staining with α-actinin and Myomesin. **(C)** Co-localization of Fibin and α-actinin at the sarcomeric z-disc in murine heart tissue sections. **(D)** *Tbx5* mRNA expression in NRVCMs overexpressing Fibin (*n* = 12 per group, two independent experiments, two-tailed Student´s t-test), detected by qPCR. **(E)** Effect of Fibin on SRF-dependent gene expression in absence or presence of RhoA (*n* = 17–18 per condition, three independent experiments, Kruskal-Wallis one-way analysis of variance). **(F)** Effect of Fibin on NFAT-dependent gene expression in absence or presence of constitutively active variant of calcineurin (*n* = 17-18 per condition, three independent experiments, Kruskal-Wallis one-way analysis of variance).

To further characterise possible functions of Fibin in cardiomyocytes we aimed to uncover Fibin’s localization within NRVCMs and in heart tissue sections of adult WT mice. Immunostainings of Fibin revealed a colocalization of Fibin with α-actinin, a well-known protein of the cardiac z-disc (Figure 3A). We did not detect an overlap with an antibody against the sarcomeric m-band protein Myomesin (Figure 3A). To exclude unspecific antibody binding we confirmed these results by creating an adenovirus coding for a fusion protein of Fibin and C-terminal green fluorescent protein (GFP). As expected, infected NRVCMs showed a strong fluorescent signal located at the cardiac z-disc (Figure 3B). The co-localization of Fibin and α-actinin was also detectable in heart tissue sections of adult mice (Figure 3C).

In zebrafish it has been shown that Fibin modulates the expression of *Tbx5* (Wakahara et al., 2007). Tbx5 is known as a transcription factor which regulates SRF-dependent gene expression via its interaction with Myocardin (Wang et al., 2011). Based on these facts we hypothesized that the anti-hypertrophic effect of Fibin could be transmitted through modulation of Tbx5 expression and consequently SRF-dependent signalling. Indeed, qPCR analysis revealed a downregulation of *Tbx5* in NRVCMs overexpressing Fibin (Figure 3D). To examine inhibitory effects of Fibin on RhoA-SRF-signalling we performed SRF response element driven luciferase reporter assays in the absence or presence of RhoA, an endogenous activator of SRF signalling. Fibin effectively inhibited SRF-dependent gene expression (Figure 3E) both in the absence and presence of the upstream activator RhoA.

Regarding the upregulation of Fibin in calcineurin-transgenic mice and its localization at the sarcomeric z-disc, we wondered whether Fibin overexpression also affects calcineurin-NFAT-signalling. By performing NFAT-luciferase reporter assays we observed also a dose-dependent reduction of the NFAT-reporter activity by Fibin overexpression in the presence of constitutively active calcineurin (Figure 3F).

Taken together, our *in vitro* analysis revealed Fibin as a novel negative regulator of cardiomyocyte hypertrophy through inhibition of both, SRF- and NFAT-dependent signalling.

### 3.4 Fibin transgenic mice develop a dilated cardiomyopathy accompanied by systolic dysfunction with age

**FIGURE 4:**
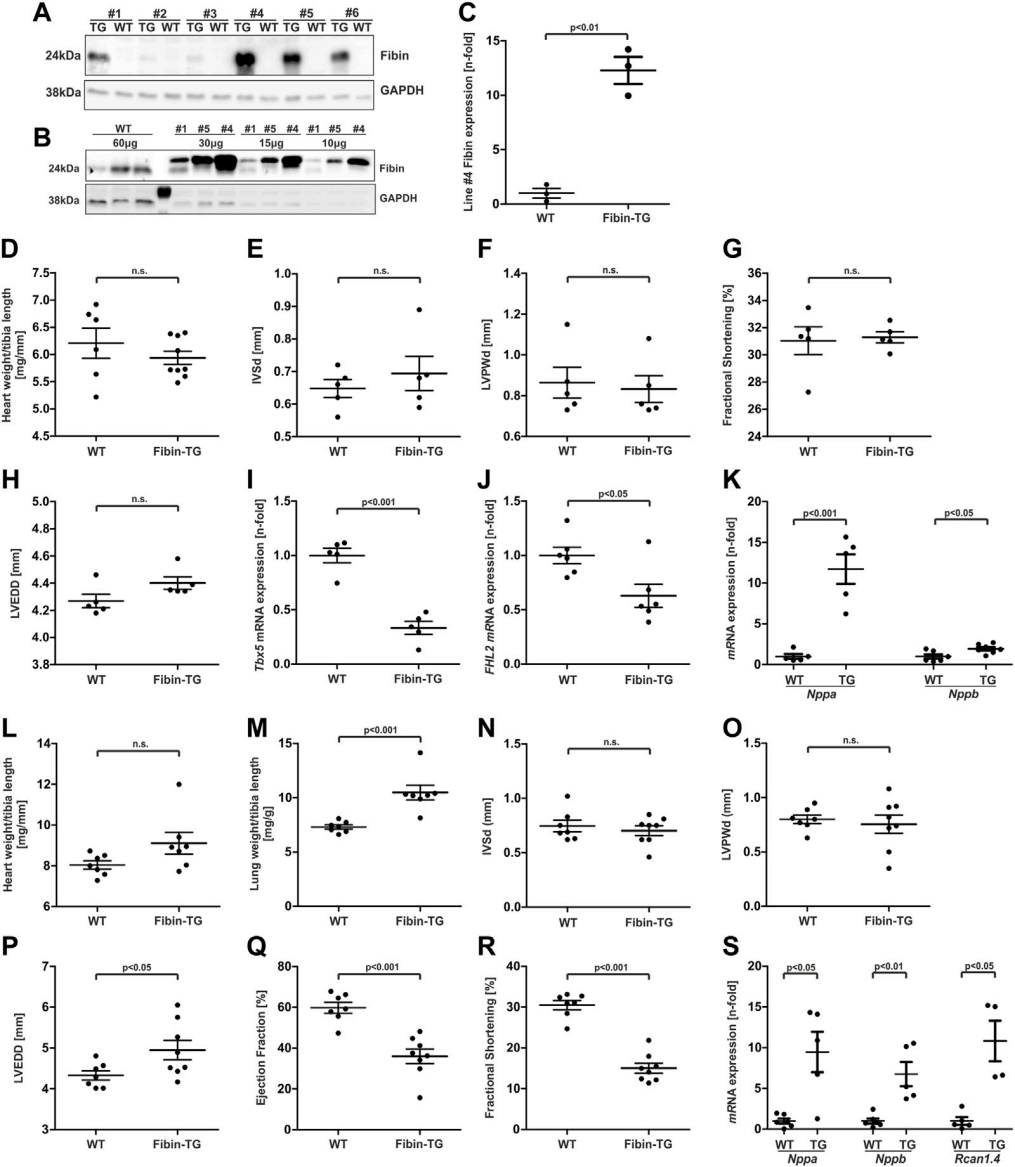
Basic characterization of mice with cardiac-specific overexpression of Fibin. **(A and B)** Transgenic overexpression of Fibin in different founder lines detected by Western blot. Lines #1, #4 and #5 were selected for further analyses. In this figure the data are presented for male animals of line #4 with the highest Fibin expression. For the basic characterization of female mice and transgenic lines #1 and #5 see Supplementary Material. Analyses of Fibin-TG mice at the age of 8 weeks: **(C)** Quantification of overexpression of Fibin in Fibin-TG mice of line #4 detected by Western blot, **(D)** heart weight to tibia length ratio (WT *n* = 6, Fibin-TG *n* = 9), **(E)** interventricular septum thickness at end-diastole (IVSd, *n* = 5), **(F)** left ventricular posterior wall thickness at end-diastole (LVPWd, *n* = 5), **(G)** fractional shortening (*n* = 5), **(H)** left ventricular end diastolic diameter diameter (LVEDD) (n = 5), **(I)** *Tbx5* mRNA expression (n = 5) detected by qPCR, **(J)** *FHL2* mRNA expression (n = 6) detected by qPCR and **(K)** *Nppa* (n = 5) and *Nppb* (*n* = 6–7) mRNA expression detected by qPCR. **(L-S)** Analyses of male Fibin-TG mice at the age of 6 months: **(L)** Heart weight to tibia length ratio (n = 7), **(M)** lung weight to tibia length ratio (*n* = 7), **(N)** interventricular septum thickness at end-diastole (IVSd, WT *n* = 7, Fibin-TG n = 8), **(O)** left ventricular posterior wall thickness at end-diastole (LVPWd, WT *n* = 7, Fibin-TG *n* = 8), **(P)** LVEDD (WT *n* = 7, Fibin-TG *n* = 8), **(Q)** ejection fraction (WT *n* = 7, Fibin-TG *n* = 8), **(R)** fractional shortening (WT *n* = 7, Fibin-TG *n* = 8), and **(S)** *Nppa* (*n* = 5-6)*, Nppb* (*n* = 5–6) and *Rcn1.4* (*n* = 4–5) mRNA expression detected by qPCR. Statistical significances were calculated by two-tailed Student´s *t*-test.

To validate the anti-hypertrophic effect of Fibin *in vivo* we generated a transgenic mouse model that overexpresses Fibin under the control of an α-MHC promoter (Gulick et al., 1991b). Western blot analysis of heart tissue of these transgenic (TG) founders revealed protein expression with different levels of Fibin overexpression (Figures 4A,B). We chose male animals of the line with the highest expression levels (#4, 12.3-fold, Figure 4C) for further analyses. At the age of 8 weeks TG mice did not show signs of cardiac hypotrophy or hypertrophy (Figures 4D–F). Echocardiographic analyses of cardiac function and left ventricular dimensions of TG animals revealed no differences compared to wildtype (WT) littermates (4G-H In line with our *in vitro* findings, *Tbx5* and *FHL2*, an established target gene of SRF (Philippar et al., 2004), was strongly downregulated in Fibin TG mice (Figures 4I,J). Surprisingly, Fibin overexpression led to a strong induction of members of the hypertrophic gene program, like *Nppa* and *Nppb* (Figure 4K).

To test if this subclinical hypertrophic signalling might become apparent as a distinct phenotype with age, we also characterised Fibin-TGs at the age of 6 months. Indeed, TG mice displayed a heart failure phenotype without significant cardiac hypertrophy (Figure 4L, N and O), but increased lung weights suggesting pulmonary congestion (Figure 4M), and echocardiographically left ventricular dilation (+14.37%, Figure 4P). Moreover, echocardiography of TG animal revealed cardiac dysfunction. The ejection fraction as well as fractional shortening were significantly reduced in Fibin-TG mice compared to their WT littermates (Figure 4Q–R). Again, Fibin overexpression led to an induction of *Nppa*, *Nppb* and additionally of *Rcan1.4* (Figure 4S). Analyses of TG lines with lower Fibin overexpression (#5, #1) showed similar results. For the whole data set of morphological and echocardiographic analyses of all transgenic mouse lines at the age of 8 weeks and 6 months, see Supplementary Tables S2–S9.

### 3.5 Fibin overexpression promotes heart failure induced by pressure overload

**FIGURE 5:**
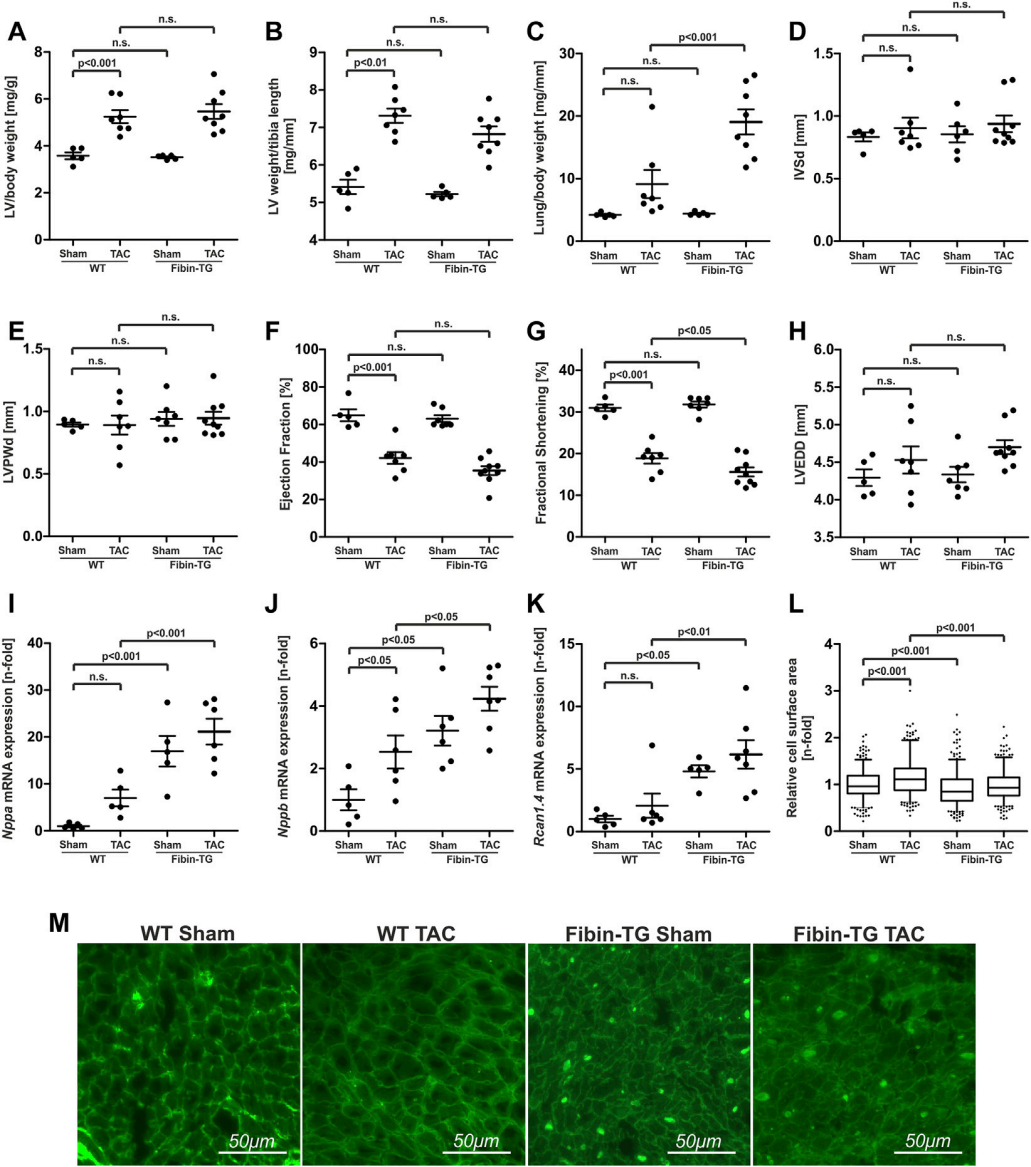
Transgenic overexpression of Fibin in mice promotes cardiac dysfunction after transverse aortic constriction (TAC). Morphological analyses of male Fibin-TG mice after TAC at the age of 8 weeks: **(A)** Left ventricular (LV) weight to body weight ratio, **(B)** LV weight to tibia length ratio, **(C)** lung weight to body weight ratio (WT sham n = 5, WT TAC *n* = 7, Fibin-TG sham *n* = 5, Fibin-TG TAC *n* = 8). Echocardiographic analyses of male Fibin-TG mice 2 weeks after TAC at the age of 8 weeks: **(D)** Interventricular septum thickness at end-diastole (IVSd), **(E)** left ventricular posterior wall thickness at end-diastole (LVPWd), **(F)** ejection fraction, **(G)** fractional shortening, **(H)** left ventricular end diastolic diameter (LVEDD) (WT sham *n* = 5, WT TAC *n* = 7, Fibin-TG sham n = 6-7, Fibin TG TAC *n* = 9). **(I)** *Nppa* mRNA expression levels (WT sham n = 5, WT TAC *n* = 5, Fibin-TG sham *n* = 5, Fibin-TG TAC *n* = 6) **(J)** *Nppb* mRNA expression levels (WT sham *n* = 5, WT TAC *n* = 6, Fibin-TG sham *n* = 6, Fibin-TG TAC *n* = 7) and **(K)** *Rcan.1.4* mRNA expression levels (WT sham *n* = 5, WT TAC *n* = 6, Fibin-TG sham *n* = 5, Fibin-TG TAC *n* = 7) in Fibin-TG mice after TAC detected by qPCR. **(L)** Cell surface area (CSA) of single cardiomyocytes of Fibin-TG mice after TAC (WT sham *n* = 367, WT TAC *n* = 358, Fibin-TG sham *n* = 373, Fibin-TG TAC *n* = 367). **(M)** Representative images of lectin stained tissue sections for the measurement of CSA of single cardiomyocytes. Statistical significances were calculated by two-way ANOVA followed by Student-Newman-Keuls *post hoc* test.

Given the anti-hypertrophic effect of Fibin *in vitro*, we expected an attenuation of the hypertrophic response in young Fibin-TG mice under conditions of pathological cardiac hypertrophy. To test this hypothesis we subjected Fibin-TG mice to pressure overload by transverse aortic constriction (TAC). We chose male 8 weeks old animals, because they did not show signs of cardiac dysfunction at this age. The transverse aorta was constricted for a period of 2 weeks. Sham operated mice served as a control. As intended, TAC led to severe cardiac hypertrophy and impaired cardiac function in WT mice. Surprisingly, Fibin-TG mice after TAC showed no differences compared to banded wildtype mice in terms of left ventricular hypertrophy (Figures 5A,B, Figure 5D–E) but a significant increase of lung weight to body weight ratio (*n* = five to eight per group, Figure 5C) indicating more pronounced heart failure compared to WT mice after TAC. Additional echocardiographic analysis revealed only marginally differences between Fibin-TG and WT mice after TAC. While no significant difference in ejection fraction (Figure 5F) and left ventricular enddiastolic diameter (Figure 5H) were observed, the fractional shortening was slightly decreased in Fibin-TG mice (Figure 5G). In line with the data of basic characterisation, Fibin-TG mice showed elevated expression levels of *Nppa, Nppb* and *Rcan1.4* already under baseline conditions (Figures 5I–K)*.*

Since Fibin overexpression results in a dramatic reduction of cell size in isolated cardiomyocytes upon hypertrophic stimulation, we wondered if Fibin overexpression *in vivo* has an influence on the CSA of single cardiomyocytes. To test this, we performed lectin staining and subsequent CSA measurements in tissue sections of WT and Fibin-TG mice. Interestingly, Fibin overexpression reduced the TAC-induced increase in CSA of cardiomyocytes by 18% compared to banded WT animals (Figure 5L), which contradicts a mere cardiomyocyte hypertrophy. Of note, lectin staining revealed another remarkable finding. Consistently, in all sections of Fibin-TG mice, we found numerous crude deposits (Figure 5M). For the whole data set of morphological and echocardiographic analyses, see Supplementary Tables S9, S10.

### 3.6 Fibin-transgenic mice develop severe cardiac dysfunction by overexpression of calcineurin

Because our *in vitro* studies revealed inhibition of calcineurin/NFAT-dependent signalling by Fibin we decided to test the effect of Fibin overexpression also in transgenic mice with heart-specific overexpression of a constitutively active form of calcineurin. Calcineurin transgenic mice (CnA-TG) develop severe cardiac hypertrophy and heart failure (Molkentin et al., 1998). We crossbred Fibin-TG mice with calcineurin-TG mice and characterized male animals at the age of 6 weeks.

**FIGURE 6:**
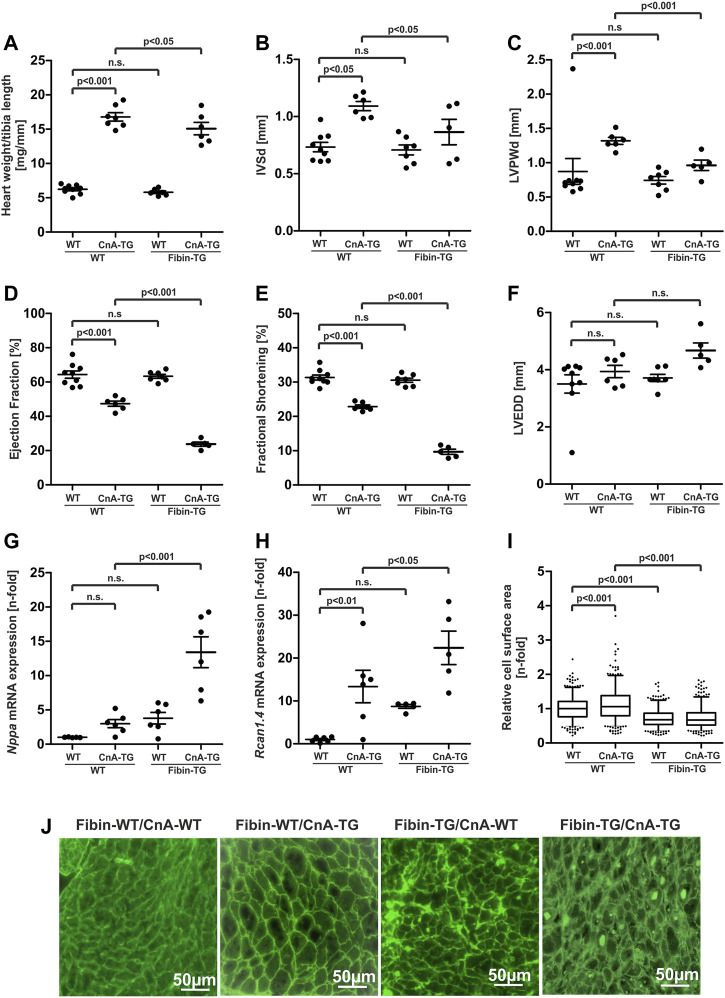
Fibin overexpression in Calcineurin-TG mice attenuates cardiac hypertrophy but promotes heart failure. Characterisation of male Fibin-TG and Calcineurin-TG crossbreeds at the age of 6 weeks. **(A)** Heart weight to tibia length ratios of Fibin-TG and Calcineurin-TG (CnA-TG) crossbreeds (WT *n* = 9, CnA-TG *n* = 7, Fibin-TG *n* = 6, Fibin-TG/CnA-TG *n* = 6). Echocardiographic analyses of Fibin-TGxCnA-TG crossbreeds: **(B)** Interventricular septum thickness at end-diastole (IVSd), **(C)** left ventricular posterior wall thickness at end-diastole (LVPWd), **(D)** ejection fraction, **(E)** fractional shortening, **(F)** left ventricular end diastolic diameter (LVEDD) (WT n = 9, CnA-TG *n* = 6, Fibin-TG *n* = 7, Fibin-TG/CnA-TG *n* = 5). **(G)** *Nppa* mRNA expression levels (WT *n* = 5, CnA-TG *n* = 6, Fibin-TG *n* = 6, Fibin-TG/CnA-TG n = 6) and **(H)** *Rcan1.4* mRNA expression levels (WT *n* = 6, CnA-TG *n* = 6, Fibin-TG *n* = 5, Fibin-TG/CnA-TG *n* = 5) in Fibin-TGxCnA-TG crossbreeds, detected by qPCR. **(I)** Cell surface area (CSA) of single cardiomyocytes in Fibin-TGxCnA-TG crossbreeds (WT *n* = 397, CnA-TG *n* = 384, Fibin-TG *n* = 362, Fibin-TG/CnA-TG *n* = 388). **(J)** Representative images of lectin stained tissue sections for the measurement of CSA of single cardiomyocytes. Statistical significances were calculated by two-way ANOVA followed by Student-Newman-Keuls *post hoc* test.

Morphological analysis revealed cardiac hypertrophy induced by calcineurin in both groups, but with different severity. Within the group of calcineurin-TG mice, overexpression of Fibin led to significant lower heart weight to tibia length ratios (−10.19%, *p* < 0.05, Figure 6A) as well as to reduced thickness of the interventricular septum (−20.79%, *p* < 0.05, Figure 6B) and the left ventricular posterior wall (−27.07%, *p* < 0.001, Figure 6C). Next, we wondered if this attenuation of Calcineurin-induced hypertrophy is beneficial for cardiac function. As expected, Calcineurin overexpression led to impaired systolic function with reduced ejection fraction and fractional shortening (Figures 6D,E). Surprisingly, double-transgenic mice exhibited even worse systolic function with dramatically lower ejection fraction (23.77% ± 2.03% vs 47.35% ± 1.85%, *p* < 0.001, *n* = 5-9 per group) and fractional shortening (9.7% ± 0.8% vs 22.87% ± 0.73%, *p* < 0.001, *n* = 5-9 per group) On molecular level, double-transgenic mice showed excessively increased *Nppa* expression levels (Figure 6G) and elevated *Rcan 1.4* expression levels (Figure 6H). However, Fibin-TG mice did not develop hypertrophy of single cardiomyocytes (Figures 6I,J). On the contrary, Fibin-TG mice showed reduced CSA at baseline conditions and no induction of CSA through Calcineurin. Again, crude deposits were present in all sections of Fibin-TG mice (Figure 6J). For the whole data set of morphological and echocardiographic analyses, see Supplementary Tables S12, S13.

Taken together, the anti-hypertrophic effect of Fibin in cultured cardiomyocytes was reproducible, at least microscopically, in mice under conditions of pathological hypertrophy. This should have beneficial effects regarding cardiac function and progression to heart failure. However, Fibin transgenic mice developed severe systolic dysfunction under Calcineurin overexpression. At the age of 6 months, even unstressed mice developed a dilated cardiomyopathy with severe contractile dysfunction. We assumed that deposits that are detected by lectin staining might cause the unexpected phenotype.

### 3.7 Protein aggregates, increased endoplasmic reticulum (ER) stress, unfolded protein response (UPR) and UPR-mediated apoptosis are responsible for cardiomyopathy phenotype of Fibin-TG mice

**FIGURE 7:**
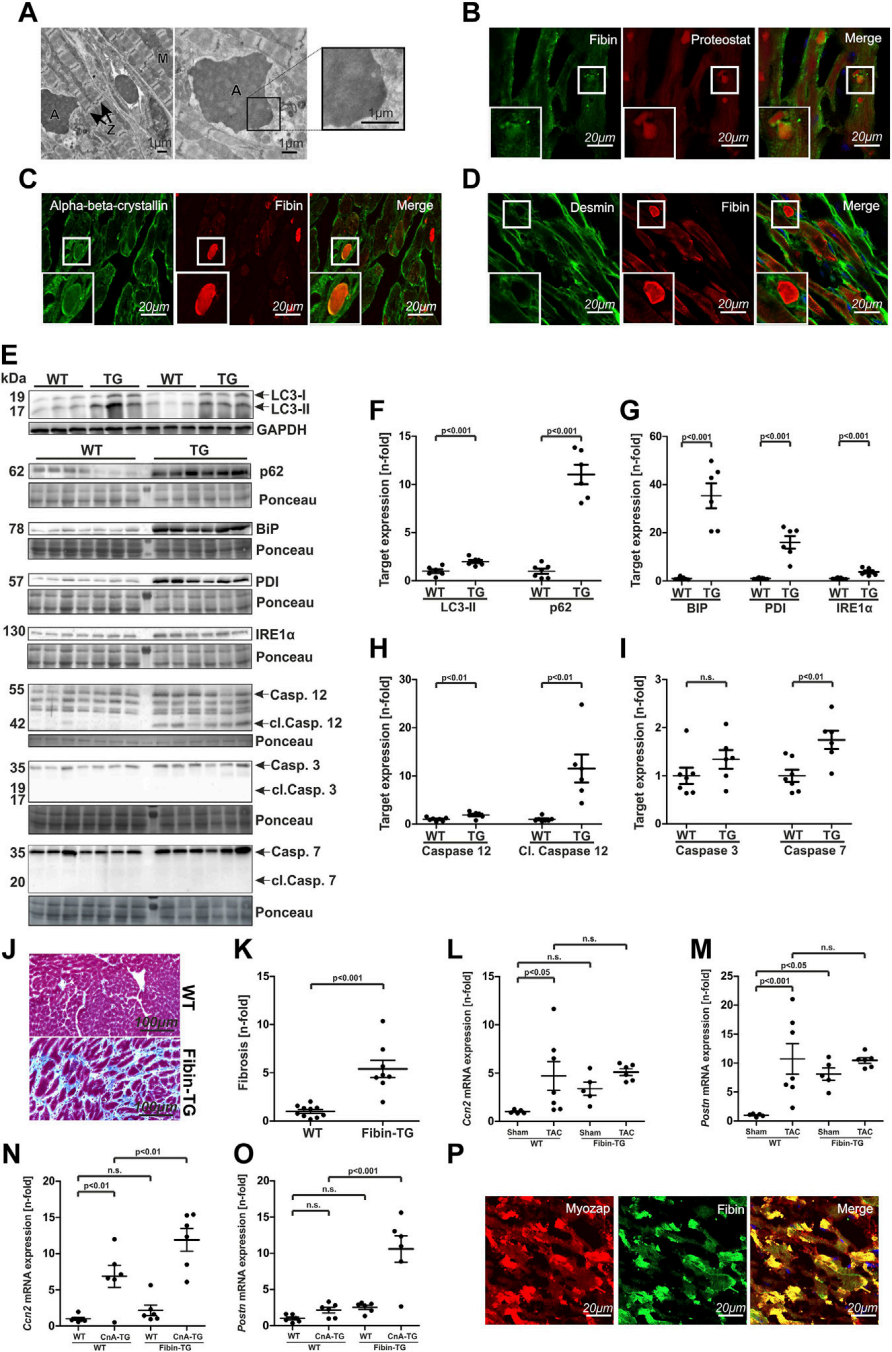
Fibin overexpression in mice leads to protein-aggregate-associated cardiomyopathy with induction of unfolded protein response (UPR), UPR-mediated apoptosis, autophagy and cardiac fibrosis. **(A)** Electron microscopy of heart tissue sections of Fibin-TG mice. A: Aggregate, M: Mitochondria, Z: Z-disc. **(B–D)** Immunostaining of heart tissue sections of Fibin-TG mice with Proteostat fluorescence dye, antibodies against Fibin, αβ-crystallin and Desmin. **(E–I)** Western blot analysis of proteins involved in autophagy, unfolded protein response (UPR) and UPR-mediated apoptosis in Fibin-TG and wildtype mice: Protein levels of **(F)** LC3-II and p62, **(G)** BIP, PDI and IRE1α, **(H)** Caspase 12 and cleaved Caspase (WT *n* = 7, TG *n* = 6), **(I)** Caspase 3 and Caspase 7 (WT *n* = 7, TG *n* = 6). LC3-II is normalized to GAPDH, all other proteins are normalized to whole protein content stained by Ponceau. **(J and K)** Masson trichrome staining of heart tissue sections of Fibin-TG mice and quantification of myocardial fibrosis (WT *n* = 10, Fibin-TG *n* = 8).**(L)** and **(M)** *Ccn2* and *Postn* mRNA expression levels in male Fibin-TG mice after TAC at the age of 8 weeks measured by qPCR. **(N)** and **(O)** *Ccn* and *Postn* mRNA expression levels in Fibin-TGxCnA-TG crossbreeds at the age of 6 weeks measured by qPCR. **(P)** Immunostainings of Fibin and Myozap on heart tissue sections of Myozap-TG and wildtype mice. Except panel **(L–O)** all experiments were performed with heart tissue sections or protein samples of male Fibin-TG mice at the age of 8 weeks. Statistical significances were calculated by two-tailed Student´s *t*-test or two-way ANOVA followed by Student-Newman-Keuls *post hoc* test.

Electron microscopy of heart tissue sections confirmed the presence of crude intracellular deposits of variable size in Fibin-TG mice. These aggregates appeared electron-dense without distinct reference to any cell organelle. Of note, the sarcomere including the z-disc seems to be intact in transgenic mice (Figure 7A).

Immunostaining with Proteostat detection dye (Shen et al., 2011) identified the deposits as protein aggregates (Figure 7B).

In cardiac disease, accumulation of protein aggregates is primarily known in the context of myofibrillar myopathies (MFMs). In these diseases, mutations in z-disc or z-disc related proteins lead to protein aggregation and subsequently to muscle weakness and cardiomyopathy. To check for similarities to MFMs we performed immunostaining experiments in heart tissue sections of Fibin-TG mice with antibodies against the most prominent proteins involved in aggregate formation in MFMs, Desmin and αΒ-crystallin. Besides Fibin, we identified αΒ-crystallin (CRYAB) to be present in the aggregates (Figure 7C) but not Desmin (Figure 7D).

In MFMs aggregation of misfolded proteins leads to increased autophagic activity (Zheng et al., 2011). To test if autophagy is also induced in Fibin-TG mice we quantified the expression levels of the autophagy markers LC3-II and p62. Both of them were significantly upregulated, which is a typical constellation in cardiac proteinopathy (Zheng et al., 2011) (Figures 7E,F).

In other models of protein aggregate associated diseases aggregate formation leads to endoplasmic reticulum stress (ER stress) and subsequently to an activation of the unfolded protein response (UPR) (Roussel et al., 2013). To test whether UPR is activated in Fibin-TG mice, we quantified the expression levels of several key members of the UPR signalling cascade via Western blot. These experiments revealed a robust induction of BiP, PDI and less pronounced IRE1α (Figures 7E,G).

It is established, that prolonged ER stress and subsequent UPR signalling are potent inducers of cardiomyocyte apoptosis (Dorn, 2009). Especially activation of caspase 12, an ER-localized caspase, is crucial for initiation of ER-mediated apoptosis (Nakagawa et al., 2000). To test if the activation of UPR is capable of inducing ER-mediated apoptosis in Fibin-TG mice, we analysed the expression levels of caspase 12 as well as of the effector caspases 3 and 7. Indeed, caspase 12 as well as cleaved caspase 12 was significantly upregulated (Figures 7E,H). While the expression level of caspase 3 was not affected, caspase 7 was also upregulated in Fibin-TG mice. However, the activated, cleaved forms of both could not be detected (Figures 7E,I).

Another hallmark of cardiac remodelling is the replacement of apoptotic cardiomyocytes by fibrous tissue. Masson trichrome staining and subsequent quantification of fibrosis revealed dramatically increased levels of fibrosis in Fibin-TG mice (Figures 7J,K). For confirmation, we measured the mRNA expression levels of *Ccn2* (Cellular communication network factor 2) and *Postn* (Periostin), two established markers of cardiac fibrosis (Maruyama and Imanaka-Yoshida, 2022), in Fibin-TG mice after TAC and in Fibin-TGxCnA-TG crossbreeds. *Ccn2* is significantly induced in WT TAC and CnA-TG mice (Figures 7L,N). In double transgenic mice *Ccn2* is even higher (Figure 7N). In Fibin-TG mice that underwent TAC we did not detect significantly higher levels (Figure 7L). *Periostin* showed highest levels in Calcineurin and Fibin double transgenic mice which supports the notion that Fibin overexpression is associated with fibrosis (Figure 7O).

Previously, it has been shown that Myozap transgenic mice develop a protein-aggregate-associated cardiomyopathy with dysregulation of UPR signalling, induction of autophagy and apoptosis (Frank et al., 2014). To check whether Fibin is involved in aggregate formation in this specific mouse model with a similar phenotype, we performed immunostaining experiments in heart tissue sections of Myozap-TG mice. Indeed, Fibin was highly enriched in the protein aggregates of Myozap-TG mice (Figure 7P).

## 4 Discussion

Here we show that Fibin plays a role in cardiomyocyte hypertrophy and cardiomyopathy. Fibin is expressed in several adult tissues including skeletal and heart muscle (Lakner et al., 2011). Yet, so far little was known about its function in the heart, except that it is induced in right ventricles of rats during chronic pulmonary embolism (Zagorski et al., 2009).

### 4.1 Fibin co-localizes with α-actinin at the sarcomeric z-disc in cardiomyocytes

The important role of the sarcomeric z-disc regarding mechanical integrity and force transmission in cardiac and skeletal muscle is well established. Beyond its mechanical role, the cardiac z-disc has recently been identified as a nodal point in hypertrophic signalling as many signalling molecules involved in hypertrophic cascades are localized to the cardiac z-disc (Frank et al., 2006; Frank and Frey, 2011). Here we show that in cardiomyocytes, Fibin also co-localizes at least partially with α-actinin at the sarcomeric z-disc.

In earlier studies, Fibin was suggested to be a secreted protein because Fibin’s sequence displays a predicted N-terminal signal sequence for endoplasmic reticulum entry and a glycosylation site at Asn30 (Lakner et al., 2011). In line with this, immunostaining experiments in COS-7 cells revealed a subcellular localization of Fibin in the endoplasmic reticulum. Though Fibin was detected in the supernatant of cultivated COS-7 cells that overexpress Fibin (Wakahara et al., 2007), the proof of a relevant secretion of Fibin from COS-7 cells failed (Lakner et al., 2011). The presence of Fibin in the supernatant of COS-7 cells seems to be caused by cell damage rather than by secretion of intact cells. The localization of Fibin in NRVCMs at the sarcomeric z-disc indicates that Fibin has different subcellular localizations and thus likely diverse functions in different cell types. Of note, this is not an uncommon feature. In a recently published comprehensive study, Thul et al. used immunofluorescence microscopy to map 12,003 human proteins in 30 cellular compartments and substructures (Thul et al., 2017). Their data revealed that the majority of the analysed proteins show a distribution in more than one cellular department. Presumably, in cardiomyocytes a yet unknown mechanism prevents Fibin translocation to the endoplasmatic reticulum and thus its way through the secretory pathway.

### 4.2 Fibin is a novel negative regulator of cardiomyocyte hypertrophy via NFAT- and SRF signalling

Our study reports for the first time a distinct function of Fibin in mammalian cells. Fibin is upregulated in different mouse models of cardiac hypertrophy and, of note, in patients with dilated cardiomyopathy, implicating clinical relevance. Our *in vitro* studies revealed a strong anti-hypertrophic effect as Fibin was sufficient to prevent cardiomyocyte hypertrophy induced by PE. While knockdown of Fibin is not sufficient to cause cellular hypertrophy under baseline conditions, it induces the expression of hypertrophic genes and exaggerates the hypertrophic response upon biochemical stimulation. Based on these findings, we concluded that Fibin might be part of a counter-regulatory mechanism in cardiac hypertrophy.

We found two signalling pathways that mediate cardiac hypertrophy were repressed by Fibin. On the one hand, Fibin overexpression diminishes SRF-dependent gene expression in NRVCMs through downregulation of *Tbx5*. Tbx5 is known as a transcription factor, which regulates SRF-dependent gene expression via its interaction with Myocardin (Wang et al., 2011), a member of the Myocardin-related transcription factors (MRTF) family. It has been shown earlier that inhibition of Tbx5 prevents cardiomyocyte hypertrophy (Wang et al., 2017).

In earlier studies, Fibin was suggested to induce the expression of *Tbx5*, because in zebrafish embryos the expression of Fibin temporally preceded the expression of *Tbx5* and a knockdown of Fibin abolished the expression of *Tbx5* (Wakahara et al., 2007). Up to now, we can only speculate about the reasons for these seemingly contradictory findings. Differences between species and developmental stages might be possible explanations. The alpha-MHC promoter is active at rather low levels only during mouse heart development (Ng et al., 1991). Therefore, the overexpression of Fibin might not be sufficient to cause developmental abnormalities by Tbx5 dysregulation in our mouse model.

On the other hand, overexpression of Fibin in cardiomyocytes effectively inhibited NFAT-dependent gene expression. Of note, this inhibition persisted after overexpression of a constitutively active form of calcineurin, indicating that Fibin inhibits either calcineurin directly or the pathway downstream of calcineurin. Interestingly, Fibin overexpression was still sufficient to reduce cardiomyocyte size in the presence of overexpressed constitutively active calcineurin *in vivo*. However, the effect on macroscopic hypertrophy seems to be overruled by development of a protein-aggregate-associated cardiomyopathy.

The inhibition of SRF- as well as NFAT-dependent gene expression by Fibin overexpression suggests that Fibin might serve as nodal point that mediates a crosstalk between RhoA-SRF-signalling and Calcineurin-NFAT-signalling. It has been shown earlier that these two signalling pathways interact, because RhoA itself is capable to activate Calcineurin in a calcium-independent way (Rajapurohitam et al., 2012). Furthermore, Calcineurin is at least partially involved in the activation of SRF-dependent gene expression, e.g., in DT40 cells, a chicken B-cell line (Hao et al., 2003).

### 4.3 Fibin overexpression in transgenic mice leads to a protein-aggregate-associated cardiomyopathy and promotes progression to heart failure under conditions of pathological cardiac hypertrophy

The anti-hypertrophic effect of Fibin is, at least partially, still detectable in Fibin-TG mice as Fibin overexpression abrogated the increase in cell surface area of cardiomyocytes induced by TAC or Calcineurin overexpression. Consistent with our *in vitro* data, the expression of Tbx5 is diminished in Fibin-TG mice. However, with rising age, even unstressed mice developed a dilated cardiomyopathy with severe contractile dysfunction. Fibin overexpression in mice is not protective in terms of hypertrophy, as expected based on our findings in isolated cardiomyocytes, but rather promotes heart failure under conditions of pressure overload or Calcineurin overexpression.

A possible cause for this unexpected phenotype of Fibin transgenic mice could be the accumulation of large intracellular protein aggregates. In other models of protein aggregate associated diseases, aggregate formation leads to endoplasmic reticulum stress (ER stress) and subsequently to an activation of the unfolded protein response (UPR) (Roussel et al., 2013; Frank et al., 2014). The UPR comprises complex signalling pathways leading to translational attenuation, transcriptional activation of chaperone genes and activation of ER-associated degradation (ERAD) (Groenendyk et al., 2010). Excessive or prolonged UPR leads to apoptosis. A broad range of cardiovascular diseases is accompanied by increased UPR (Zhang et al., 2019). Here we show that the protein aggregates in Fibin-TG mice lead to strong activation of the UPR and subsequently to an induction of the ER-associated caspase 12 as well as of caspase 7. However, the lack of cleaved caspase 3 and 7 supports the notion that the common pathway of apoptosis is not activated in these animals. We analysed protein content of caspases in relatively young Fibin TG mice at the age of 6 weeks in order to detect early effects of Fibin overexpression that are not influenced by other pathways involved in heart failure, the obvious phenotype of aged Fibin TG mice. One can speculate that apoptosis might be activated by prolonged ER stress to a later time point.

Furthermore, we could show increased levels of fibrosis in Fibin-TG mice. Fibrotic reorganization is a central hallmark of cardiac remodelling finally resulting in heart failure. Likely, this effect overrules the potentially beneficial antihypertrophic effect of Fibin and contributes to the development of dilated cardiomyopathy in Fibin-TG.

Of note, our study was performed in C57BL/6N mice, which show a nonadaptive response of wildtype mice on TAC. Accordingly, we observed marked cardiac hypertrophy and left ventricular dysfunction. In contrast, C57BL6/J mice develop compensatory cardiac hypertrophy with less pronounced deterioration of LV function and dilatation (Garcia-Menendez et al., 2013). Thus, it would also be interesting to study the effect of Fibin overexpression on cardiac hypertrophy in C57BL6/J mice after TAC.

### 4.4 Protein aggregates in Fibin-TG mice: A link to myofibrillar myopathies?

Accumulation of protein aggregates is a common feature of a group of chronic neuromuscular diseases, termed myofibrillar myopathies (MFMs) (Claeys and Fardeau, 2013). Myofibrillar myopathies are genetic muscle disorders leading to progressive muscle weakness, cardiomyopathy and/or peripheral neuropathy (Batonnet-Pichon et al., 2017). MFMs are associated with mutations in genes that encode for z-disc proteins, including Desmin, Myotilin, Filamin C, and αB-crystallin (Batonnet-Pichon et al., 2017). Histological and ultrastructural analysis of muscle biopsies from patients suffering from myofibrillar myopathies and animal models revealed disorganization of the interfibrillar network beginning at the sarcomeric z-disc and protein aggregation as common histological features of MFMs (Batonnet-Pichon et al., 2017). Interestingly, these protein aggregates do not only consist of the mutant protein itself. Immunohistochemical and mass spectrometry analyses of aggregates revealed several other z-disc proteins but also cytosolic and even extracellular matrix proteins (Selcen et al., 2004; Kley et al., 2013; Maerkens et al., 2016). Hence, the process of aggregate formation seems to be more complex than simple deposition of individual aggregate-prone proteins (Maerkens et al., 2016).

Our findings in Fibin-TG mice resembles the phenotype of MFMs in several major points. Like the majority of proteins involved in aggregate formation in MFMs, Fibin is a z-disc protein. Moreover, we could show that the aggregates do not consist of Fibin only but also include αΒ-crystallin (CRYAB), a member of the family of small heat shock proteins. It has been shown earlier that mutations in CRYAB itself can cause myofibrillar myopathy and cardiomypathy (Vicart et al., 1998; Selcen and Engel, 2003). Furthermore αΒ-crystallin is found in protein aggregates of patients suffering from MFMs caused by mutations in other z-disc proteins, for example, in desminopathy (Maerkens et al., 2013), filaminopathy (Kley et al., 2013) or myotilinopathy (Maerkens et al., 2016). Additionally we could show, that autophagic activity is increased in Fibin-TG mice, a common feature of MFMs (Zheng et al., 2011).

Finally, we found Fibin in protein aggregates of Myozap-TG mice, another mouse model of protein-aggregate-associated cardiomyopathy (Frank et al., 2014), also demonstrating that aggregate formation in Fibin-TG mice is not just due to an unspecific aggregation of overexpressed protein.

Our data suggest that Fibin-TG mice might serve as a model to provide insight into aggregate formation in the context of myofibrillar myopathies. Currently, there is no pharmacological treatment available for patients suffering from myofibrillar myopathies. Uncovering the underlying mechanisms of aggregate formation could help to identify specific targets, for a pharmacological treatment.

## Data Availability

The original contributions presented in the study are included in the article/Supplementary Materials, further inquiries can be directed to the corresponding author.
