# Supplementary material for: Fibin regulates cardiomyocyte hypertrophy and causes protein-aggregate-associated cardiomyopathy in vivo
Source: Front Mol Biosci. 2023 Jun 5;10:1169658. doi: 10.3389/fmolb.2023.1169658 (PMC10278231; doi:10.3389/fmolb.2023.1169658)
Supplement: Supplementary file 1 [file DataSheet1.PDF]

Supplementary Material

Title: Fibin regulates cardiomyocyte hypertrophy and causes protein-aggregate-associated cardiomyopathy *in vivo*

Matthias Petersen, Nesrin Schmiedel, Franziska Dierck, Susanne Hille, Anca Remes, Frauke Senger, Inga Schmidt, Renate Lüllmann-Rauch, Oliver J. Müller, Derk Frank, Ashraf Y. Rangrez, Norbert Frey[*,†], and Christian Kuhn[†]

***Correspondence:**
Norbert Frey, norbert.frey@med.uni-heidelberg.de

1 Supplementary Figures

Supplementary figure 1

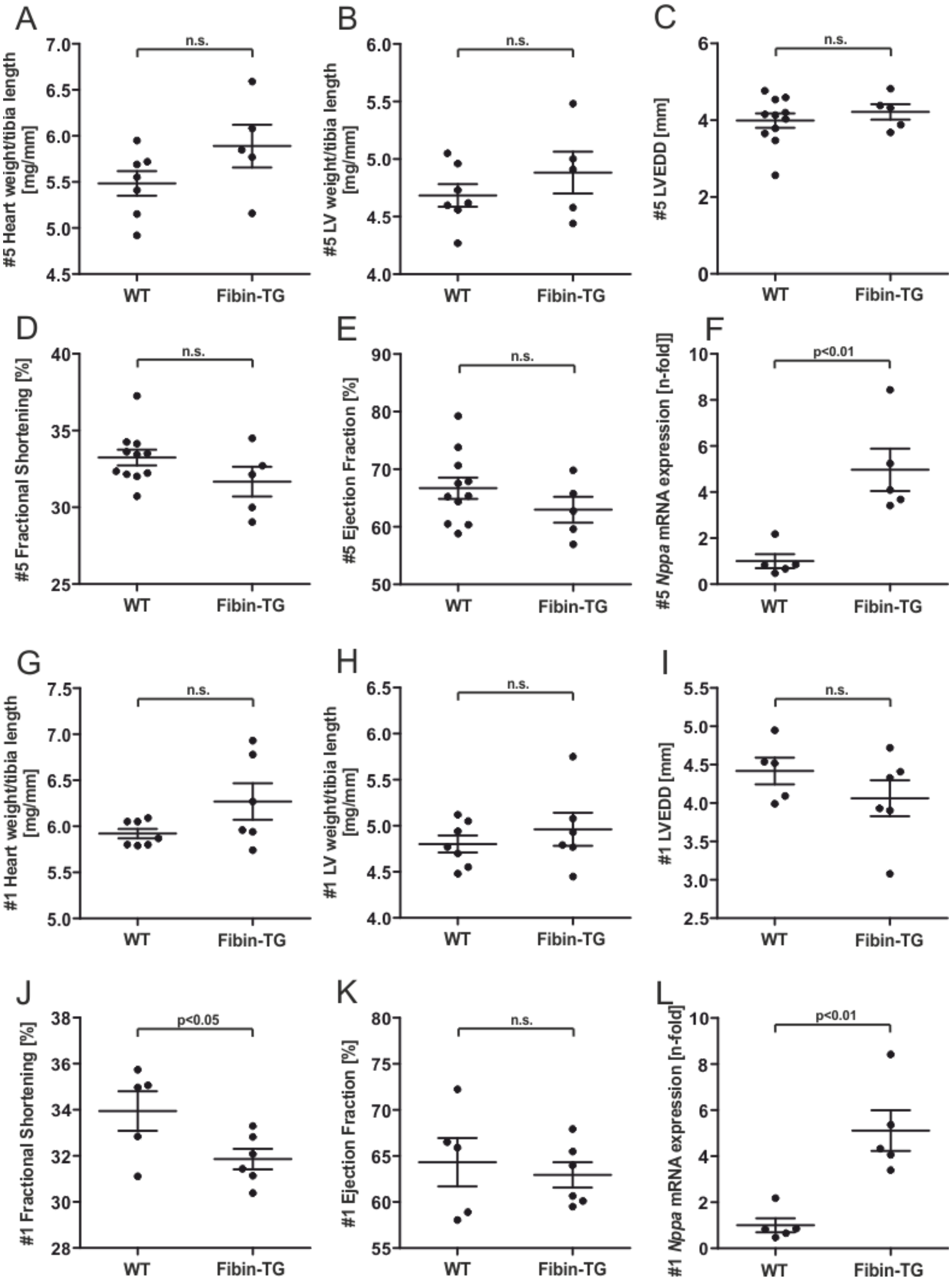

Supplementary figure 1: Basic characterisation of mice with cardiac-specific overexpression of Fibin.
Basic characterization of Fibin transgenic mouse line #5 at the age of 8 weeks: (**A**) Heart weight to tibia length ratio (WT n=7, Fibin-TG n=5), (**B**) left ventricular weight to tibia length ratio (WT n=7, Fibin-TG n=5), (**C**) left ventricular end diastolic diameter diameter (LVEDD) (WT n=11, Fibin-TG n=5), (**D**) fractional shortening (WT n=11, Fibin-TG n=5), (**E**) ejection fraction (WT n=11, Fibin-TG n=5), (**F**) *Nppa* mRNA expression (n=5) measured by qPCR. Basic characterisation of Fibin transgenic mouse line #1: (**G**) Heart weight to tibia length ratio (WT n=7, Fibin-TG n=6), (**H**) left ventricular weight to tibia length ratio (WT n=7, Fibin-TG n=6), (**I**) LVEDD (WT n=5, Fibin-TG n=6), (**J**) fractional shortening (WT n=5, Fibin-TG n=6), (**K**) ejection fraction (WT n=5, Fibin-TG n=6), (**L**) *Nppa* mRNA expression (n=5) measured by qPCR. Statistical significances were calculated by two-tailed Student´s t-test.

2 Supplementary Tables

Supplementary Table 1: Primary and secondary antibodies. IF: Immunoflourescence, WB: Western Blot

**Target protein**	**Host**	**Company**	**Catalog Number**	**Applicati on**	**Dilution**
α-Actinin	Mouse	Sigma-Aldrich	A7811	IF	1:200
αB-crystallin	Mouse	Abcam	Ab13496	IF	1:200
BiP	Rabbit	Cell Signaling via NEB	#3177	WB	1:1000
Caspase-12	Rabbit	Cell Signaling via NEB	#2202	WB	1:1000
Caspase 3	Rabbit	Cell Signaling via NEB	#9662	WB	1:1000
Caspase 7	Rabbit	Cell Signaling via NEB	#9492	WB	1:1000
Desmin	Rabbit	Abcam	ab8592	IF	1:80

Fibin	Rabbit	Sigma-Aldrich	HPA040120	IF, WB	1:200, 1:500
Fibin	Mouse	Abcam	ab169369	IF	1:200
Fibin	Rabbit	Thermo Fisher Scientific	BS-16085R	IF	1:200
IRE1α	Rabbit	Cell Signaling via NEB	#3294	WB	1:500
LC3B	Rabbit	Cell Signaling via NEB	#2775	WB	1:1000
Myomesin	Mouse	Creative Diagnostics	DMAB9591	IF	1:200
Myozap	Mouse	Progen	651169	IF	1:10
PDI	Rabbit	Cell Signaling via NEB	#3501	WB	1:1000
SQSTM1/p62	Rabbit	Cell Signaling via NEB	#5114	WB	1:1000
**Secondary target**	**Host**	**Company**	**Catalog Number**	**Application**	**Dilution**
Rabbit IgG-HRP	Donkey	Santa Cruz	sc-2305	WB	1:10000
Mouse IgG-HRP	Goat	Santa Cruz	sc-2302	WB	1:10000
Mouse IgG-AF546	Donkey	Thermo Fisher Scientific	A10036	IF	1:1000

Rabbit IgG-AF488	Chicken	Thermo Fisher Scientific	A21441	IF	1:1000
Mouse IgG-AF488	Chicken	Thermo Fisher Scientific	A11001	IF	1:1000
Rabbit IgG-AF546	Donkey	Thermo Fisher Scientific	A10040	IF	1:1000

Supplementary Table 2: Morphological analyses of Fibin transgenic mice (Fibin-TG) at the age of 8 weeks (line#4). WT: Wildtype

	**♂WT (n=6)**	**♂Fibin-TG (n=9)**	**♀WT (n=5)**	**♀Fibin-TG (n=5)**	**♂Fibin-TG vs. ♂WT**	**♀Fibin-TG vs. ♀WT**
**Body weight [g]**	24 ±0.9	21.8 ±0.6	19.7 ±0.3	19.1 ±0.6	n.s.	n.s.
**Heart weight [mg]**	100.9 ±5.1	94.7 ±2.3	93.4 ±5.3	100.6 ±4.2	n.s.	n.s.
**Left ventricular weight [mg]**	81.8 ±4.6	78 ±2.3	73 ±3.2	70.15 ±2	n.s.	n.s.
**Tibia length [mm]**	16.2 ±0.1	15.9 ±0.1	16 ±0.1	15.9 ±0.1	n.s.	n.s.
**Heart weight/body weight [mg/g]**	4.2 ±0.2	4,3 ±0.1	4.7 ±0.2	5.3 ±0.3	n.s.	n.s.
**Left ventricular weight/ body weight [mg/g]**	3.5 ±0.2	3.6 ±0.1	3.7 ±0.1	3.7 ±0.2	n.s.	n.s.
**Heart weight/tibia length [mg/mm]**	6.2 ±0.3	5.9 ±0.1	5.8 ±0.3	6.3 ±0.3	n.s.	n.s.
**Left ventricular weight/tibia length [mg/mm]**	5.0 ±0.3	4.9 ±0.1	4.6 ±0.2	4.4 ±0.1	n.s.	n.s.
**Lung weight [mg]**	144.4 ±8.4	134.7 ±4.9	126.4 ±4.3	117.1 ±2.4	n.s.	n.s.

Supplementary Table 3: Echocardiographic analyses of Fibin transgenic mice (Fibin-TG) at the age of 8 weeks (line#4). WT: Wildtype

	**♂WT (n=5)**	**♂Fibin-TG (n=5)**	**♀WT (n=9)**	**♀Fibin-TG (n=5)**	**♂Fibin-TG vs. ♂WT**	**♀Fibin-TG vs. ♀WT**
**Heart rate [bpm]**	374.8 ±6,3	371.2 ±10	400.6 ±20.5	432.1 ±26.2	n.s.	n.s.
**Fractional Shortening [%]**	31.0 ±1	31.3 ±0.4	32.4 ±0.6	31.3 ±1	n.s.	n.s.
**Ejection Fraction [%]**	65.1 ±2.5	65.3 ±0.8	64.5 ±1.60	60.1 ±2.6	n.s.	n.s.
**Interventricular septum thickness at end-systole (IVSs) [mm]**	0.81 ±0.04	0.99 ±0.06	0.93 ±0.04	0.96 ±0.07	n.s.	n.s.
**Interventricular septum thickness at end-diastole (IVSd) [mm]**	0.65 ±0.03	0,69 ±0.05	0.66 ±0.03	0.67 ±0.07	n.s.	n.s.
**Left ventricular internal dimension at end-systole (LVEDs) [mm]**	3.3 ±0.1	3.3 ±0.1	2.9 ±0.2	3.1 ±0.2	n.s.	n.s.
**Left ventricular internal dimension at end-diastole (LVEDd) [mm]**	4.3 ±0.05	4.4 ±0.05	3.9 ±0.2	4 ±0.2	n.s.	n.s.
**Left ventricular posterior wall thickness at end-systole (LVPWs) [mm]**	1.02 ±0.06	1.05 ±0.06	0.98 ±0.03	0.9 ±0.11	n.s.	n.s.
**Left ventricular posterior wall thickness at end-diastole (LVPWd) [mm]**	0.86 ±0.08	0.83 ±0.07	0.78 ±0.04	0.72 ±0.09	n.s.	n.s.

Supplementary Table 4: Morphological analyses of Fibin transgenic mice (Fibin-TG) at the age of 6 months (line#4). WT: Wildtype

	**♂WT N=7**	**♂Fibin-TG N=7**	**WT vs. ♂Fibin-TG**
**Body weight [g]**	30.4±1.1	29.3±1.1	n.s.
**Heart weight [mg]**	137.6±4.7	155.1±10.8	n.s.
**Left ventricular weight [mg]**	106±3.1	98.4±6.2(n=6)	n.s.
**Tibia length [mm]**	17.1±0.2	17±0.2	n.s.
**Heart weight/body weight [mg/g]**	4.5±0	5.4±0.5	n.s.
**Left ventricular weight/ body weight [mg/g]**	3.5±0.1	3.3±0.1 (n=6)	n.s.
**Heart weight/tibia length [mg/mm]**	8.0±0.2	9.1±0.5	n.s.
**Left ventricular weight/tibia length [mg/mm]**	6.2±0.1	5.8±0.3 (n=6)	n.s.
**Lung weight [mg]**	153.4±3.3	177.7±11.2	n.s.

Supplementary Table 5: Echocardiographic analyses of Fibin transgenic mice (Fibin-TG) at the age of 6 months (line#4). WT: Wildtype

	**♂WT N=7**	**♂Fibin-TG N=8**	**WT vs. ♂Fibin-TG**
**Heart rate [bpm]**	423.5±16.1	357.5±11.7	p<0.05
**Fractional Shortening [%]**	30.5±1.1	15±1.2	p<0.001
**Ejection Fraction [%]**	59.8±2.7	36±3.6	p<0.001
**Interventricular septum thickness at end-systole (IVSs) [mm]**	0.96±0.06	1.11±0.23	n.s.
**Interventricular septum thickness at end-diastole (IVSd) [mm]**	0.75±0.05	0.7±0.05	n.s.
**Left ventricular internal dimension at end-systole (LVEDs) [mm]**	3.6±0.1	3.9±0.3	n.s.
**Left ventricular internal dimension at end-diastole (LVEDd) [mm]**	4.3±0.1	5±0,2	p<0.05
**Left ventricular posterior wall thickness at end-systole (LVPWs) [mm]**	0.91±0.03	1.2±0.13	n.s.
**Left ventricular posterior wall thickness at end-diastole (LVPWd) [mm]**	0.8±0.04	0.76±0.8	n.s.

Supplementary Table 6: Morphological analyses of Fibin transgenic mice (Fibin-TG) at the age of 8 weeks (line#5). WT: Wildtype

	**♂WT (n=7)**	**♂Fibin-TG (n=5)**	**♀WT (n=7)**	**♀Fibin-TG (n=9)**	**♂Fibin-TG vs. ♂WT**	**♀Fibin-TG vs. ♀WT**
**Body weight [g]**	23.1 ±0.3	23 ±0.7	19.5 ±0.5	19.1 ±0.3	n.s.	n.s.
**Heart weight [mg]**	88.6 ±2.3	95.2 ±4	84.3 ±4.1	87.4 ±2	n.s.	n.s.
**Left ventricular weight [mg]**	75.7 ±1.6	78.8 ±3	69.8 ±4 (n=6)	70.2 ±1.7	n.s.	n.s.
**Tibia length [mm]**	16.2 ±0	16.2 ±0.1	16.1 ±0.1	15.8 ±0.1	n.s.	n.s.
**Heart weight/body weight [mg/g]**	3.9 ±0.1	4.1 ±0.1	4.3 ±0.1	4.6 ±0.1	n.s.	n.s.
**Left ventricular weight/ body weight [mg/g]**	3.3 ±0.1	3.4 ±0.1	3.6 ±0.1 (n=6)	3.7 ±0	n.s.	n.s.
**Heart weight/tibia length [mg/mm]**	5.5 ±0.1	5.9 ±0.2	5.2 ±0.2	5.5 ±0.1	n.s.	n.s.
**Left ventricular weight/tibia length [mg/mm]**	4.7 ±0.1	4.9 ±0.2	4.3 ±0.2 (n=6)	4.4 ±0.1	n.s.	n.s.
**Lung weight [mg]**	137.4 ±4	131.7 ±4.8	131.8 ±4.3	128 ±3	n.s.	n.s.

Supplementary Table 7: Echocardiographic analyses of Fibin transgenic mice (Fibin-TG) at the age of 8 weeks (line#5). WT: Wildtype

	**♂WT (n=11)**	**♂Fibin-TG (n=5)**	**♀WT (n=6)**	**♀Fibin-TG (n=5)**	**♂Fibin-TG vs. ♂WT**	**♀Fibin-TG vs. ♀WT**
**Heart rate [bpm]**	412.4 ±13	404.9 ±15	377.3 ±13.9	363.8 ±17.4	n.s.	n.s.
**Fractional Shortening [%]**	35.7 ±0.7	34.4 ±1.6	37.5 ±3.4	34.3 ±1.4	n.s.	n.s.
**Ejection Fraction [%]**	65.4 ±1	63.6 ±2	67.3 ±3.8	63.4 ±1.9	n.s.	n.s.
**Interventricular septum thickness at end-systole (IVSs) [mm]**	1.07 ±0.05	1.08 ±0.05	0.97 ±0.06	0.94 ±0.1	n.s.	n.s.
**Interventricular septum thickness at end-diastole (IVSd) [mm]**	0.75 ±0.04	0,72 ±0.03	0.74 ±0.06	0.64 ±0.05	n.s.	n.s.
**Left ventricular internal dimension at end-systole (LVEDs) [mm]**	2.7 ±0.2	3 ±0.2	2.5 ±0.2	2.7 ±0.2	n.s.	n.s.
**Left ventricular internal dimension at end-diastole (LVEDd) [mm]**	4 ±0.2	4.2 ±0.2	3.5 ±0.2	3.7 ±0	n.s.	n.s.
**Left ventricular posterior wall thickness at end-systole (LVPWs) [mm]**	1.22 ±0.06	1.04 ±0.05	1.06 ±0.06	1 ±0.08	n.s.	n.s.
**Left ventricular posterior wall thickness at end-diastole (LVPWd) [mm]**	0.93 ±0.04	0.85 ±0.07	0.87 ±0.08	0.86 ±0.05	n.s.	n.s.

Supplementary Table 8: Morphological analyses of Fibin transgenic mice (Fibin-TG) at the age of 8 weeks (line#1). WT: Wildtype

	**♂WT (n=8)**	**♂Fibin-TG (n=6)**	**♀WT (n=8)**	**♀Fibin-TG (n=6)**	**♂Fibin-TG vs. ♂WT**	**♀Fibin-TG vs. ♀WT**
**Body weight [g]**	23.6 ±0.5	24.1 ±0.7	19.4 ±0.4	19.1 ±0.4	n.s.	n.s.
**Heart weight [mg]**	97 ±1.8	102.2 ±3.4	79.3 ±2.2	84.5 ±1.3	n.s.	n.s.
**Left ventricular weight [mg]**	78.3 ±1.5	83.6 ±3.7	65.5 ±1.9	70 ±1.8	n.s.	n.s.
**Tibia length [mm]**	16.2 ±0.1	16.3 ±0.1	15.8 ±0.1	15.6 ±0.1	n.s.	n.s.
**Heart weight/body weight [mg/g]**	4.1 ±0.1	4.2 ±0.1	4.1 ±0.1	4.4 ±0.1	n.s.	$p<0.05$
**Left ventricular weight/ body weight [mg/g]**	2.9 ±0.4	3.5 ±0.1	3.4 ±0.1	3.7 ±0.1	n.s.	$p<0.05$
**Heart weight/tibia length [mg/mm]**	6 ±0.1	6.3 ±0.2	5 ±0.1	5.4 ±0.1	n.s.	$p<0.05$
**Left ventricular weight/tibia length [mg/mm]**	4.2 ±0.6	5.1 ±0.2	4.1 ±0.1	4.5 ±0.1	n.s.	n.s.
**Lung weight [mg]**	130.1 ±6	136.7 ±3.4	127 ±1.6	124 ±2.9	n.s.	n.s.

Supplementary Table 9: Echocardiographic analyses of Fibin transgenic mice (Fibin-TG) at the age of 8 weeks (line#1). WT: Wildtype

	**♂WT (n=5)**	**♂Fibin-TG (n=6)**	**♀WT (n=5)**	**♀Fibin-TG (n=5)**	**♂Fibin-TG vs. ♂WT**	**♀Fibin-TG vs. ♀WT**
**Heart rate [bpm]**	420.4 ±20.8	393.5 ±17.1	371.5 ±7.7	384.2 ±19.6	n.s.	n.s.
**Fractional Shortening [%]**	35.5 ±0.9	36 ±1	32.5 ±1.7	36.6 ±1.1	n.s.	n.s.
**Ejection Fraction [%]**	64.9 ±1.2	65.8 ±1.4	60.7 ±2.6	67 ±1.4	n.s.	n.s.
**Interventricular septum thickness at end-systole (IVSs) [mm]**	0.98 ±0.08	0.98 ±0.07	0.94 ±0.05	0.88 ±0.03	n.s.	n.s.
**Interventricular septum thickness at end-diastole (IVSd) [mm]**	0.71 ±0.06	0,72 ±0.04	0.74 ±0.05	0.73 ±0.02	n.s.	n.s.
**Left ventricular internal dimension at end-systole (LVEDs) [mm]**	3.3 ±0.1	2.8 ±0.2	2.9 ±0.2	2.8 ±0.1	n.s.	n.s.
**Left ventricular internal dimension at end-diastole (LVEDd) [mm]**	4.4 ±0.2	4.1 ±0.2	3.8 ±0.2	3.6 ±0.1	n.s.	n.s.
**Left ventricular posterior wall thickness at end-systole (LVPWs) [mm]**	1.03 ±0.05	1.15 ±0.09	0.97 ±0.06	1.02 ±0.05	n.s.	n.s.
**Left ventricular posterior wall thickness at end-diastole (LVPWd) [mm]**	0.85 ±0.02	0.89 ±0.06	0.89 ±0.08	0.85 ±0.02	n.s.	n.s.

Supplementary Table 10: Morphological analyses of male Fibin transgenic mice (Fibin-TG) at after transverse aortic constriction (TAC) the age of 8 weeks. WT: Wildtype

	**WT Sham (n=5)**	**WT TAC (n=7)**	**Fibin-TG Sham (n=5)**	**Fibin-TG TAC (n=8)**	**WT Sham vs. WT TAC**	**WT Sham vs. Fibin-TG Sham**	**WT TAC vs. Fibin-TG TAC**
**Body weight [g]**	25.1 ±1	22.6 ±0.8	24.1 ±1	20.6 ±0.8	n.s.	n.s.	n.s.
**Heart weight [mg]**	115 ±4.5	146.8 ±3.8	116.7 ±4.5	174.1 ±3.6	p<0.001	n.s.	p<0.001
**Left ventricular weight [mg]**	89.7 ±3.6	116.8 ±3	84.6 ±3.6	110.9 ±2.8	p<0.001	n.s.	n.s.
**Tibia length [mm]**	16.6 ±0.2	16 ±0.2	16.2 ±0.2	16.2 ±0.2	n.s.	n.s.	n.s.
**Heart weight/body weight [mg/g]**	4.6 ±0.4	6.6 ±0.4	4.9 ±0.4	8.6 ±0.4	p<0.01	n.s.	p=0.001
**Left ventricular weight/ body weight [mg/g]**	3.6 ±0.3	5.2 ±0.2	3.5 ±0.3	5.5 ±0.2	p<0.001	n.s.	n.s.
**Heart weight/tibia length [mg/mm]**	6.9 ±0.3	9.2 ±0.3	7.2 ±0.3	10.7 ±0.2	p<0.001	n.s.	p<0.001
**Left ventricular weight/tibia length [mg/mm]**	5.4 ±0.2	7.3 ±0.2	5.2 ±0.2	6.8 ±0.2	p<0.001	n.s.	n.s.
**Lung weight [mg]**	136.1 ±31.5	194.7 ±26.6	135.1 ±31.5	382 ±24.9	n.s.	n.s.	p<0.001

Supplementary Table 11: Echocardiographic analyses of male Fibin transgenic mice (Fibin-TG) after transverse aortic constriction (TAC) at the age of 8 weeks. WT: Wildtype

	**WT Sham (n=5)**	**WT TAC (n=7)**	**Fibin-TG Sham (n=7)**	**Fibin-TG TAC (n=9)**	**WT Sham vs. WT TAC**	**WT Sham vs. Fibin-TG Sham**	**WT TAC vs. Fibin-TG TAC**
**Heart rate [bpm]**	388.3 ±13.2	390.3 ±11.2	377.1 ±11.2	369.8 ±10.4 (n=8)	n.s.	n.s.	n.s.
**Fractional Shortening [%]**	31 ±1.3	18.9 ±1.1	31.8 ±1.1	15.6 ±0.9	p<0.001	n.s.	p<0.05
**Ejection Fraction [%]**	64.9 ±3.1	42.1 ±2.6	63.1 ±1,4	35.4 ±2.3	p<0.001	n.s.	n.s.
**Interventricular septum thickness at end-systole (IVSs) [mm]**	1.1 ±0.12	1.08 ±0.1	1.29 ±0.1	1.17 ±0.09	n.s.	n.s.	n.s.
**Interventricular septum thickness at end-diastole (IVSd) [mm]**	0.83 ±0.08	0.91 ±0.07	0.85 ±0.07 (n=6)	0.94 ±0.06	n.s.	n.s.	n.s.
**Left ventricular internal dimension at end-systole (LVEDs) [mm]**	3.2 ±0.2	3.8 ±0.2	3.4 ±0.2	4.1 ±0.1	p<0.05	n.s.	n.s.
**Left ventricular internal dimension at end-diastole (LVEDd) [mm]**	4.3 ±0.1	4.5 ±0.1	4.3 ±0.1	4.7 ±0.1	n.s.	n.s.	n.s.
**Left ventricular posterior wall thickness at end-systole (LVPWs) [mm]**	1.06 ±0.05	1.1 ±0.04	1.07 ±0.04	1.1 ±0.04	n.s.	n.s.	n.s.
**Left ventricular posterior wall thickness at end-diastole (LVPWd) [mm]**	0.9 ±0.07	0.89 ±0.06	0.94 ±0.06	0.95 ±0.05	n.s.	n.s.	n.s.

Supplementary Table 12: Morphological analyses of male Fibin transgenic (Fibin-TG) and Calcineurin transgenic (CnA-TG) crossbreeds at the age of 6 weeks. WT: Wildtype

	**WT/ WT (n=9)**	**WT/ CnA-TG (n=7)**	**Fibin -TG/ WT (n=6)**	**Fibin-TG/ CnA-TG (n=6)**	**WT/ WT vs. WT/ CnA-TG**	**WT/ WT vs. Fibin-TG/ WT**	**WT/ CnA-TG vs. Fibin-TG/ CnA-TG**
**Body weight [g]**	19.5 ±0.5	19.2 ±0.6	18.9 ±0.6	15.5 ±0.6	n.s.	n.s.	p<0.001
**Heart weight [mg]**	94.4 ±5.7	248.9 ±6.5	85,3 ±7	209.1 ±7	p<0.001	n.s.	p<0,001
**Left ventricular weight [mg]**	73.2 ±5.4	159 ±6.2	62.5 ±6.7	110.5 ±6.7	p<0.001	n.s.	p<0.001
**Tibia length [mm]**	15.1 ±0.2	14.8 ±0.2	14.7 ±0.2	13.9 ±0.2	n.s.	n.s.	p<0.01
**Heart weight/body weight [mg/g]**	4.85 ±0.3	12.9 ±0.3	4.5 ±0.4	13.5 ±0.4	p<0,001	n.s.	n.s.
**Left ventricular weight/ body weight [mg/g]**	3.7 ±0.3	8.3 ±0.4	3.3 ±0.4	7.1 ±0.4	p<0.001	n.s.	p<0.05
**Heart weight/tibia length [mg/mm]**	6.2 ±0.5	16.8 ±0.5	5.8 ±0.6	15.1 ±0.6	p<0.001	n.s.	p<0.05
**Left ventricular weight/tibia length [mg/mm]**	4.8 ±0.4	10.8 ±0.5	4.3 ±0.5	7.99 ±0.5	p<0.001	n.s.	p<0.001
**Lung weight [mg]**	114.1 ±4.3	130.2 ±4.8	110.1 ±5.2	113.5 ±5.2	p<0.05	n.s.	p<0.05

Supplementary Table 13: Echocardiographic analyses of male Fibin transgenic (Fibin-TG) and Calcineurin transgenic (CnA-TG) crossbreeds at the age of 6 weeks. WT: Wildtype

	**WT/ WT (n=9)**	**WT/ CnA-TG (n=6)**	**Fibin -TG/ WT (n=7)**	**Fibin-TG/ CnA-TG (n=5)**	**WT/ WT vs. WT/ CnA-TG**	**WT/ WT v.s. Fibin-TG/ WT**	**WT/ CnA-TG vs. Fibin-TG/ CnA-TG**
**Heart rate [bpm]**	380.8 ±5.5	351.8 ±6.8	388.5 ±8	284.6 ±5.1	n.s.	n.s.	n.s.
**Fractional Shortening [%]**	31.3 ±0.6	22.9 ±0.7	30.5 ±0.7	9.7 ±0.8	p<0.001	n.s.	p<0.001
**Ejection Fraction [%]**	64.3 ±1.5	47.3 ±1.8	63.3 ±1.7	23.8 ±2	p<0.001	n.s.	p<0.001
**Interventricular septum thickness at end-systole (IVSs) [mm]**	1.07 ±0.07	1.37 ±0.08	1.1 ±0.08	0.84 ±0.09	p<0.05	n.s.	p<0.01
**Interventricular septum thickness at end-diastole (IVSd) [mm]**	0.73 ±0.05	1.09 ±0.06	0.71 ±0.06	0.87 ±0.07	p<0.001	n.s.	p<0.05
**Left ventricular internal dimension at end-systole (LVEDs) [mm]**	2.9 ±0.1	3.2 ±0.2	2.6 ±0.2	4.4 ±0.2	n.s.	n.s.	p<0.001
**Left ventricular internal dimension at end-diastole (LVEDd) [mm]**	3.5 ±0.2	3.9 ±0.3	3.7 ±0.3	4.7 ±0.3	n.s.	n.s.	n.s.
**Left ventricular posterior wall thickness at end-systole (LVPWs) [mm]**	0.9 ±0.05	1.43 ±0,04	0.92 ±0.05	1.02 ±0.06	p<0.001	n.s.	p<0.001
**Left ventricular posterior wall thickness at end-diastole (LVPWd) [mm]**	0.87 ±0.12	1.32 ±0.14	0.74 ±0.13	0.96 ±0.16	p<0.05	n.s.	n.s.
